# Evaluation of TOCSY mixing for sensitivity-enhancement in solid-state NMR and application of 4D experiments for side-chain assignments of the full-length 30 kDa membrane protein GlpG

**DOI:** 10.1007/s10858-024-00454-7

**Published:** 2025-01-22

**Authors:** Carl Öster, Veniamin Chevelkov, Adam Lange

**Affiliations:** 1 Research Unit Molecular Biophysics, Leibniz-Forschungsinstitut für Molekulare Pharmakologie, Robert- Rössle-Straße 10, 13125 Berlin, Germany; 2 Institut für Biologie, Humboldt-Universität zu Berlin, Invalidenstraße 42, 10115 Berlin, Germany

## Abstract

Chemical shift assignments of large membrane proteins by solid-state NMR experiments are challenging. Recent advancements in sensitivity-enhanced pulse sequences, have made it feasible to acquire ^1^H-detected 4D spectra of these challenging protein samples within reasonable timeframes. However, obtaining unambiguous assignments remains difficult without access to side-chain chemical shifts. Drawing inspiration from sensitivity-enhanced TOCSY experiments in solution NMR, we have explored the potential of ^13^C- ^13^C TOCSY mixing as a viable option for triple sensitivity-enhanced 4D experiments aimed at side-chain assignments in solid-state NMR. Through simulations and experimental trials, we have identified optimal conditions to achieve uniform transfer efficiency for both transverse components and to minimize undesired cross-transfers. Our experiments, conducted on the 30 kDa membrane protein GlpG embedded in *E. coli* liposomes, have demonstrated enhanced sensitivity compared to the most effective dipolar and J-coupling-based ^13^C- ^13^C mixing sequences. Notably, a non-uniformly sampled 4D hCXCANH spectrum with exceptionally high sensitivity was obtained in just a few days using a 600 MHz spectrometer equipped with a 1.3 mm probe operating at a magic angle spinning rate of 55 kHz.

**Supplementary Information:**

The online version contains supplementary material available at 10.1007/s10858-024-00454-7.

## Introduction

The recent development of sensitivity-enhanced transverse mixing sequences suitable for ^1^H-detected solid-state NMR experiments enables fast acquisition of multidimensional experiments for protein backbone assignments. These new sequences include two different approaches, Transverse mixing optimal control pulses (TROP) (Blahut et al. 2022, 2023) and simplified preservation of equivalent pathways spectroscopy (SPEPS) (Nimerovsky et al. 2023, 2024). Both allow for simultaneous magnetization transfers of two components, similar to the strategies previously developed for solution NMR experiments (Cavanagh and Rance 1990; Schleucher et al. 1993), which leads to a theoretical increase in the signal-to-noise ratio (SNR) by a factor of √2 per indirect dimension. It is now straight-forward to assemble all sensitivity-enhanced 3D and 4D pulse sequences required to record spectra for assignments of H, N, Cα and CO chemical shifts. However, in order to unambiguously assign challenging proteins, access to side-chain chemical shifts is critical. Total correlation spectroscopy (TOCSY) mixing was shown to be highly efficient for ^13^C- ^13^C transfers at magic angle spinning (MAS) rates of ≥ 100 kHz MAS (Stanek et al. 2016) and has since then often been applied for side-chain assignments of various types of biomolecules (Öster et al. 2018; Marchanka et al. 2018; Klein et al. 2022). The suitability of different homonuclear mixing sequences for side-chain assignments in ^1^H-detected experiments at MAS rates of 55–100 kHz has recently been thoroughly explored (Paluch et al. 2022), with the best performing sequences being dipolar recoupling enhancement through amplitude modulation (DREAM) (Verel et al. 2001), TOCSY (DIPSI-3) (Shaka et al. 1988) and Total through-bond correlation spectroscopy (TOBSY) (Baldus and Meier 1996; Baldus et al. 1997) (C9_48_^1^) (Tan et al. 2018). It is therefore useful to revisit TOCSY mixing sequences that have been shown to enable sensitivity-enhancement in solution NMR experiments (Cavanagh and Rance 1990). Here we explore the suitability of DIPSI-3 for sensitivity-enhanced solid-state NMR experiments. We first evaluate the approach by performing ^13^C-detected solid-state NMR experiments on a ^13^C^1^^5^N labelled methionine-leucine-phenylalanine (MLF) powder sample in combination with simulations of polarization transfer dynamics. Next, we show that by using DIPSI-3 for ^13^C- ^13^C mixing in triple sensitivity-enhanced 4D hCXCANH and hCXCAcoNH experiments (CX represents aliphatic carbon side-chains) we can obtain high quality spectra suitable for side-chain assignments in a challenging 30 kDa membrane protein sample, full-length GlpG, in a few days. GlpG is a rhomboid protease that cleaves its substrates within the cell membrane. We have previously investigated the structure and dynamics related to the gating mechanism and inhibitor binding in a truncated version of GlpG (GlpG_ΔN_) (Shi et al. 2019; Bohg et al. 2021, 2023; Chevelkov et al. 2023), where the cytosolic domain has been removed. Additionally, crystal structures of GlpG_ΔN_ (Wang et al. 2006; Wu et al. 2006) and solution NMR structures of the isolated cytosolic domain (Sherratt et al. 2012; Ghasriani et al. 2014) have been solved. In contrast, while we have published some first solid-state NMR spectra (Nimerovsky et al. 2024; Shi et al. 2019; Sawczyc et al. 2024) of the full length protein, it has not been characterized in detail so far. This is in part due to the complexity arising from the larger size of the protein (276 amino acids). As a first step towards the full structural and dynamic characterization of GlpG, we focus here on obtaining the chemical shift assignments of a 100% H_2_O back-exchanged ^2^H^13^C^1^^5^N–uniformly labelled protein sample in a lipid bilayer. We combine the sensitivity-enhanced TOCSY spectra with double sensitivity-enhanced 3D hCANH and hCONH and triple sensitivity-enhanced 4D hCACONH and hCOCANH spectra. The advantages of using high dimensional (≥ 4D) spectra for assignments of challenging proteins by solid-state NMR have been thoroughly explored in several studies (Klein et al. 2022; Xiang et al. 2014, 2016; Fraga et al. 2017; Zinke et al. 2017). Since we are performing the experiments on a perdeuterated sample, we mostly expect to achieve Cβ assignments from the TOCSY experiments. However, for most residue types all aliphatic carbon side-chains are available for assignments based on triple sensitivity-enhanced 4D hCXCANH and hCXCAcoNH spectra despite that the initial magnetization pool is limited to back-exchanged protons.

## Methods

### Protein expression and purification

GlpG was expressed, purified and reconstituted into liposomes (*E. coli* total lipid extract (Avanti Lipids)) as previously described (Shi et al. 2019). The protein to lipid ratio was 1:1 (w/w), corresponding to approximately 1:44 (mol/mol) and the final buffer condition for the NMR sample was 50 mM Tris-HCl, 150 mM NaCl, 1mM MgCl, pH 7.4.

## Solid-state NMR

The protein sample was packed into a 1.3 mm rotor using centrifugation at 10,000 relative centrifugal force in a benchtop centrifuge. 0.2 µl of a saturated solution of DSS in sample buffer was added to the protein sample before closing the rotor to allow for internal referencing. A thin layer of glue was added to seal the caps (see e.g. ref (Öster et al. 2024) for a detailed description of the rotor packing procedure). All solid state NMR experiments were recorded on a 600 MHz spectrometer (Bruker) equipped with a triple channel (HCN) 1.3 mm probe (Bruker) operating at a MAS rate of 55 kHz. ^1^^3^C-detected 2D hnCACX spectra were recorded on MLF using a double sensitivity-enhanced hNCACX pulse sequence modified from a previously published hNCACO pulse sequence (Blahut et al. 2023). Cross-polarization (CP) was used for ^1^H-^1^^5^N transfers, SPEPS (Nimerovsky et al. 2023) for ^1^^5^N- ^13^Cα transfers and TOCSY (DIPSI-3) (Shaka et al. 1988), TOBSY (C9_48_^1^) (Tan et al. 2018) or hSPEPS (Nimerovsky et al. 2024) for ^13^Cα- ^13^CX transfers. Experiments on GlpG were performed at a sample temperature of ca. 20 °C, based on the chemical shift of the water peak referenced internally to DSS. Standard and sensitivity-enhanced ^13^C-detected 2D hCC spectra were recorded using the different mixing sequences mentioned above. The optimal conditions for the ^13^C- ^13^C mixing sequences were determined based on ^13^C-detected 1D hncaCX and hcC spectra. 3D and 4D experiments were recorded using sensitivity-enhanced pulse sequences and ^1^H-detection. CP was used for the first magnetization transfer step (^1^H- ^13^Cα, ^1^H- ^13^CO or ^1^H- ^13^CX). TROP shapes specific for 55 kHz MAS were used for ^13^Cα-^1^^5^N, ^1^^3^CO-^1^^5^N, ^1^^3^Cα- ^13^CO (specific for 600 MHz spectrometer), ^1^^3^CO- ^13^Cα (specific for 600 MHz spectrometer) and ^15^N-^1^H transfers. DIPSI-3 was used for ^13^CX- ^13^Cα transfers. 3D hCANH and hCONH spectra were recorded in a uniform fashion and processed using TopSpin v4 (Bruker). 4D spectra were recorded using 5% non-uniform sampling (NUS). Two additional 3D hCXcaNH spectra, one with TOCSY mixing and one with hSPEPS mixing, were recorded using 25% NUS. 10 kHz Waltz-64 (Zhou et al. 2007) decoupling was applied to the ^1^H channel during acquisition of the indirect dimensions and on the ^15^N and ^13^C channels during direct ^1^H acquisition. MISSISSIPPI (Rienstra et al. 2008) was used for water suppression. Echo-Antiecho was used for all indirect dimensions in sensitivity-enhanced experiments and States-TPPI (Marion et al. 1989) was used in standard experiments. All sensitivity-enhanced pulse sequences were modified based on previously published sensitivity-enhanced pulse sequences to allow for additional dimensions to be recorded and to introduce ^13^C- ^13^C mixing for side-chain assignments. A scheme of the sensitivity-enhanced hCXCANH pulse sequence is provided in SI Fig. 1. Original pulse sequences and TROP shapes were downloaded from www.optimal-nmr.net (Blahut et al. 2022, 2023). NUS lists were generated from http://gwagner.med.harvard.edu/intranet/hmsIST (Hyberts et al. 2010, 2012). NUS spectra were reconstructed and processed using nmrPipe (Delaglio et al. 1995). Experimental details for all assignment spectra are provided in SI Table 1, and 2. Processing details for NUS spectra are provided in SI Table 3 and details for the conversion from Bruker to nmrPipe format are shown in SI Fig. 2. CCPNMR AnalysisAssign v3.1 (Skinner et al. 2016) was used for data analysis, assignments and to generate figures of multidimensional NMR spectra.

## Simulations

The software package SIMPSON 4.2.1 (Bak et al. 2000) was used to simulate polarization transfer dynamics between different components of two ^13^C spins representing Cα and Cβ nuclei for DIPSI-3, WALTZ-16 (Shaka et al. 1983), hSPEPS^CA–CB^ and C9_48_^1^ recoupling sequences. Homonuclear dipolar and scalar couplings were set to 2084.9 Hz and 35 Hz respectively. Isotropic chemical shifts for Cα and Cβ nuclei were 52 ppm and 22 ppm, while radio-frequency (RF) irradiation was applied at 37 ppm. Numerical studies were performed for 55 kHz MAS rate and 600 MHz spectrometer. The RF field strength for DIPSI-3 and WALTZ-16 was set to 10 kHz, while it was set according to the MAS rate for hSPEPS^CA–CB^ (27.5 kHz average RF strength) and C9_48_^1^ (20.625 kHz RF strength). Selected sections of the SIMPSON input file, which represent relevant computational and interaction parameters are given in the supplementary information (supplementary text). The simulations explore polarization transfer efficiency between principal components of Cα and Cβ spins, (i.e. X to X, Y to Y, Z to Z). Additionally, undesirable mixing between orthogonal magnetization components (i.e. X and Y, X and Z etc.), which, in principle, could give rise to phase distortions and/or artefacts in the spectra was systematically explored. Finally, we analysed the effects of RF inhomogeneity on the performance of the considered pulse sequences.

## Results and discussion

### Evaluation of the suitability of DIPSI-3 for sensitivity-enhanced solid-state NMR experiments

**Fig. 1:**
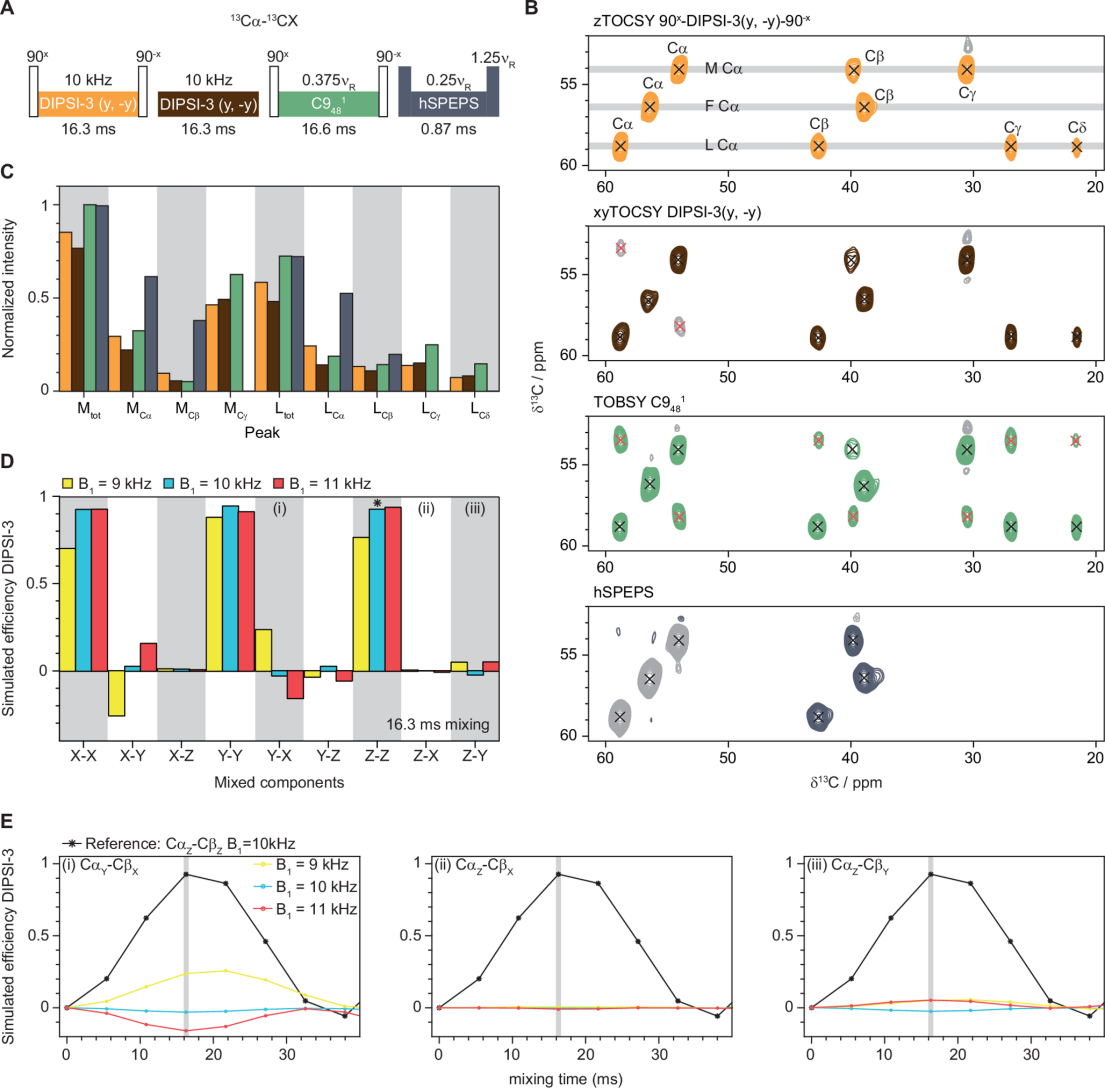
Evaluation of the suitability for sensitivity-enhancement of the DIPSI-3 mixing sequence on MLF. (**A**) Scheme of ^13^C- ^13^C mixing sequences (DIPSI-3, C9_48_^1^, and hSPEPS). (**B**) Sensitivity-enhanced 2D hnCACX spectra of ^13^C^15^N labelled MLF recorded at 55 kHz MAS on a 600 MHz spectrometer using the different ^13^C- ^13^C mixing sequences. Black crosses represent wanted peaks (assignments shown in the spectrum at the top) and red crosses represent mirrored peaks originating from unwanted cross-transfers. (**C**) Comparison of peak intensities in the spectra resulting from the different mixing sequences. The peak intensities are normalized to the highest intensity (the combined intensity for all peaks of the methionine (M_tot_) in the C9 spectrum). The colour coding is the same in panels A, B and C. (**D**) Bar plot summarizing the simulated transfer efficiencies of DIPSI-3 under the same conditions as in the experiments (16.3 ms mixing). Radio-frequency inhomogeneity is simulated by varying the nutation frequency by ± 10% from the ideal conditions (10 kHz). (**E**) Example plots representing the full simulations of DIPSI-3 for Cα_Y_ to Cβ_X_ (left, (i), Cα_Z_ to Cβ_X_ (middle, (ii) and Cα_Z_ to Cβ_Y_ (right, (iii). The grey shaded bars in E represent the values at 16.3 ms mixing, these are indicated in the bar plot in panel D ((i), (ii) and (iii). The colour coding is the same in panels D and E

To evaluate the suitability of DIPSI-3 for use in sensitivity-enhanced solid-state NMR experiments, we recorded 2D hnCACX spectra of ^13^C^15^N labelled MLF at 55 kHz MAS. DIPSI mixing sequences were designed to be strictly isotropic (Shaka et al. 1988) and are therefore expected to work well for sensitivity-enhanced experiments where the aim is to achieve equivalent magnetization transfer for two components (Cavanagh and Rance 1990). We tested two different approaches for DIPSI-3, with and without 90 degree pulses before and after the DIPSI-3 mixing. The difference between these approaches is that if we include 90 degree pulses with the phases x and -x, while the DIPSI-3 spin-lock is along y (from now on referred to as zTOCSY), the two components that are preserved are Z and X and if we don’t include the 90 degree pulses (from now on referred to as xyTOCSY) the preserved components are X and Y (see Fig. 1A for a scheme of the different mixing sequences used). Using zTOCSY (orange spectrum in Fig. 1B) with 16.3 ms mixing time at a nutation frequency of 10 kHz results in a spectrum where cross-peaks with the Cα carbons appear for all expected side-chain carbons. With the xyTOCSY approach all expected peaks also appear (brown spectrum in Fig. 1B), but some mirrored peaks with opposite phase compared to the real peaks can also be observed (grey peaks with red crosses). To further explore the appearance of mirrored peaks we recorded the same spectrum using TOBSY (C9_48_^1^) (Tan et al. 2018) (green spectrum in Fig. 1B, from now on referred to as C9) that allows for non-negligible cross-transfer (e.g. X-Y, X-Z etc.). C9 shows efficient transfer to all expected side-chains (indicated with black crosses) and additionally strong mirrored peaks (indicated with red crosses) when the spectrum is recorded in a sensitivity-enhanced fashion. In standard experiments unwanted cross-transfers can be compensated for by using a z-filter, which is typically done for applications of C9 sequences (Tan et al. 2018; Callon et al. 2023). However, applying a z-filter is not compatible with sensitivity-enhanced experiments since then only one component would be preserved. During the preparation of this manuscript a new approach using dipolar-based ^13^C- ^13^C mixing (homonuclear SPEPS) was published (Nimerovsky et al. 2024). In addition to backbone homonuclear transfers (^13^Cα- ^13^CO, ^13^CO- ^13^Cα), a mixing sequence for ^13^Cα- ^13^Cβ transfers (homonuclear SPEPS^CA−CB^, from now on referred to as hSPEPS) suitable for sensitivity-enhanced experiments was reported. We include this mixing sequence as a dipolar based alternative, instead of DREAM (Verel et al. 2001) that is not compatible with sensitivity-enhancement. As expected, hSPEPS mixing leads to a spectrum with strong Cβ-Cα cross-peaks without any mirrored peaks (dark grey spectrum in Fig. 1B). A comparison of the transfer efficiency for the different mixing sequences (Fig. 1C) reveals that hSPEPS performs best for Cα-Cβ transfers, while C9 performs best for other side-chains (slightly better than zTOCSY followed by xyTOCSY). The phenylalanine of MLF was not included in the comparison since the ^13^C offset for the indirect dimension is at the Cα chemical shift of the phenylalanine leading to that mirrored peaks will overlap with real peaks and a reliable characterization of the peak intensities is not possible. Note that hSPEPS also allows for transfer to carbons further down the side-chain if a longer mixing time is used (Nimerovsky et al. 2024), but with lower efficiency compared to TOCSY and C9 (see SI Fig. 3). The main reason for unwanted cross-transfers resulting in mirrored peaks appears to be inhomogeneity of RF pulses or rather the robustness of the mixing sequences towards RF inhomogeneity. This was realized based on SIMPSON (Bak et al. 2000) simulations of the transfer efficiencies for transfers between all possible components at different mixing times. RF inhomogeneity was tested by varying the RF power in the simulations by 10% from the ideal conditions. Figure 1D shows a bar plot summarizing the results from the simulations of DIPSI-3 and Fig. 1E shows examples of the results from the full simulations. The transfer efficiency pattern for the X and Z components are very similar (Cα_Z_ to Cβ_Z_ and Cα_X_ to Cβ_X_). The unwanted Cα_X_ to Cβ_Z_ and Cα_Z_ to Cβ_X_ cross-transfers are minimal while the Cα_X_ to Cβ_Y_ and Cα_Y_ to Cβ_X_ cross-transfers are non-negligible. This becomes clear when non-ideal RF powers are applied (red and yellow bars and lines in Fig. 1D). The simulations agree well with the experimental data regarding unwanted cross-transfers resulting in mirrored peaks, see supplementary information for the results from all simulations of DIPSI-3 (SI Fig. 4), C9_48_^1^ (SI Fig. 5) and hSPEPS (SI Fig. 6).

A comparison of the intensities of the peaks in the zTOCSY and xyTOCSY spectra (Fig. 1C) shows that the sensitivities of the Cα-CX peaks are not strongly affected by unwanted cross-transfers in MLF, although any loss of sensitivity and the appearance of mirrored peaks would be problematic for protein chemical shift assignments. Here we only tested DIPSI-3 for TOCSY mixing, but other TOCSY mixing sequences can be used provided that the magnetization transfer is equal for different components and that unwanted cross-transfers are minimal. We performed additional simulations of WALTZ-16 (SI Fig. 7) and while the unwanted cross-transfers (Cα_X_ to Cβ_Z_ and Cα_Z_ to Cβ_X_) are negligible, the transfer efficiency for different components are not equivalent. WALTZ-16 also appears to be less robust towards RF inhomogeneity compared to DIPSI-3.

### Evaluation of ^13^C- ^13^C mixing sequences on GlpG

**Fig. 2:**
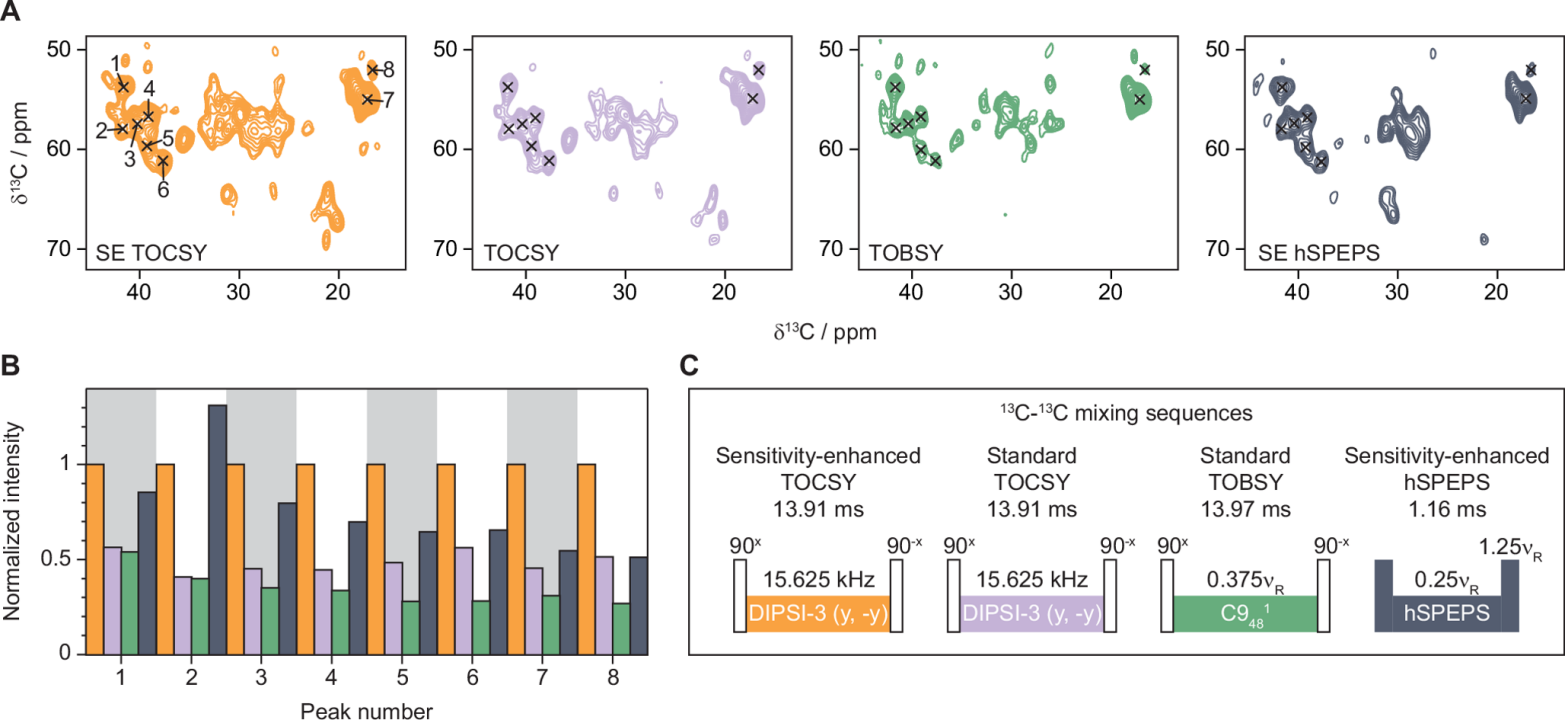
Evaluation of DIPSI-3 mixing on ^2^H^13^C^15^N labelled GlpG. (**A**) 2D hCC spectra recorded at 55 kHz MAS on a 600 MHz spectrometer using sensitivity-enhanced and standard experiments with different ^13^C- ^13^C mixing sequences. Black crosses represent peaks used for comparison. (**B**) Normalized peak intensities for 8 different peaks that can be identified in all spectra. (**C**) Schematic drawing of the ^13^C- ^13^C mixing sequences used. The same colour coding is used in all panels

Based on the experiments on MLF and the simulations of the mixing sequences, the best approach for sensitivity-enhanced solid-state NMR experiments for Cβ assignments appears to be hSPEPS and for side-chain assignments further than Cβ the zTOCSY approach (at 55 kHz MAS and on a 600 MHz spectrometer). Next we evaluated the transfer efficiency on a 100% H_2_O back-exchanged ^2^H^13^C^1^^5^N labelled GlpG sample at 55 kHz MAS. Figure 2A shows extracted regions of ^13^C-detected 2D hCC spectra using sensitivity-enhanced (using Echo-Antiecho) and standard (using States-TPPI) pulse sequences. A comparison of the peak intensities between sensitivity-enhanced and standard zTOCSY experiments (orange vs. purple in Fig. 2B) shows the expected increase in peak intensities by approximately a factor of 2 in the sensitivity-enhanced experiment, which corresponds to an increase in SNR by √2. We also compared the peak intensities to a spectrum recorded with standard acquisition using C9 for ^13^C- ^13^C mixing (green in Fig. 2) and a spectrum recorded using sensitivity-enhanced hSPEPS (dark grey in Fig. 2). For most peaks investigated in GlpG, the sensitivity-enhanced zTOCSY shows the highest intensity (Fig. 2B) and, as expected, it also gives additional peaks coming from carbons further down the side-chain compared to hSPEPS (Fig. 2A, see also SI Fig. 8 for the full spectra).

TOCSY works better than hSPEPS for GlpG, while hSPEPS works better for MLF. One possible explanation for this is that side-chain motions in GlpG averages dipolar interactions leading to less efficient dipolar recoupling. At the same time, these motions will lead to increased ^13^C *T*_*1ρ*_ relaxation times, which is beneficial for the longer TOCSY mixing sequences. MLF is a rigid powder sample with reduced dynamics compared to GlpG. The different mixing sequences may also be affected by the protonation levels of the samples. MLF is fully protonated, while GlpG is perdeuterated. At 55 kHz MAS, a higher level of protonation can result in reduced ^13^C *T*_*1ρ*_ relaxation times, potentially leading to loss of magnetization during long mixing sequences.

## Chemical shift assignment of full length GlpG

**Fig. 3:**
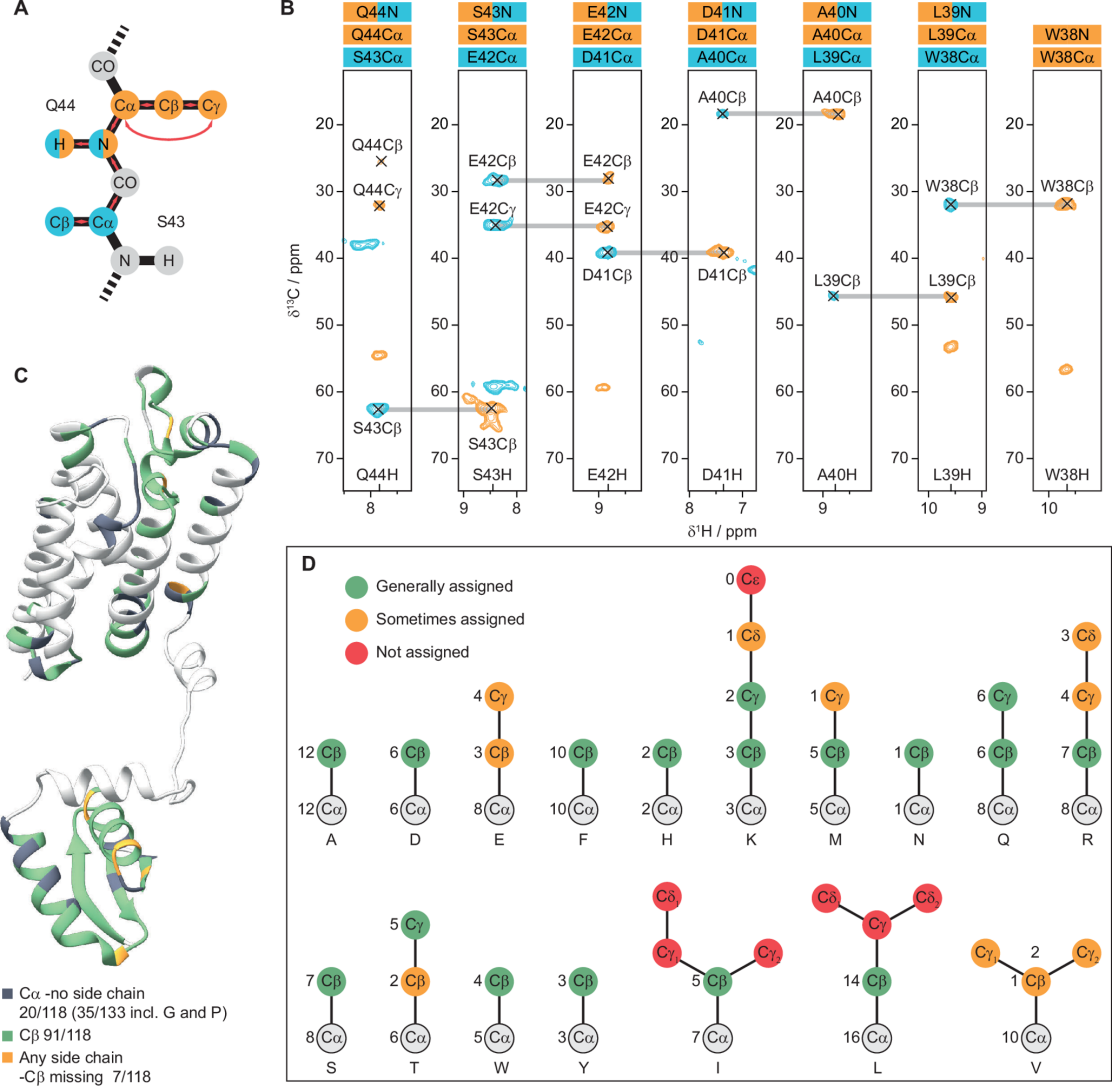
Summary of the side-chain assignments enabled by the triple sensitivity-enhanced 4D experiments. (**A**) Scheme of how the resonances are visualized in the strip plots with the magnetization transfer pathways indicated (red arrows). (**B**) Strip plots for the region Q44 to W38, showing the side-chain assignments for residue i in orange (hCxCaNH) and i-1 in blue (hCxCacoNH) connected to the backbone ^15^N and ^1^H^N^ resonances of residue i. (**C**) Summary of the side-chain assignments plotted onto a model of GlpG. Residues with Cα but no side-chain assignments are coloured in dark gray, residues with Cβ assignments are coloured in green and residues with no Cβ assignments but with other carbon side-chains assigned are coloured in orange.(**D**) Scheme summarizing the side-chain assignments for each residue type found in GlpG (except glycines and prolines). Green circles indicate side-chains that were generally assigned, orange circles represent side-chains that were sometimes assigned and red circles represent side-chains that could not be assigned.

Despite access to chemical shift assignments of the isolated transmembrane (solid-state NMR (Shi et al. 2019) and cytosolic (solution NMR (Sherratt et al. 2012; Ghasriani et al. 2014) domains, the assignment of full length GlpG (276 residues) is not trivial. Several peaks are overlapping in 3D spectra and there are many regions where the chemical shifts differ significantly between the full length protein and the isolated domains. The possibility to acquire triple sensitivity-enhanced 4D experiments, enabled by the recent development of TROP and SPEPS pulses, simplified the assignment procedure considerably. Here we used TROP for backbone ^13^C-^1^^5^N, ^1^^3^C- ^13^C and ^1^^5^N-^1^H transfers (see SI Fig. 9 for example strip plots of the backbone spectra). It should be mentioned that the TROP ^1^^5^N-^1^H transfer is less efficient compared to CP for GlpG (see SI Fig. 10 for a comparison of 1D spectra), but due to the combined sensitivity-enhancement from each indirect dimension it is still possible to record highly sensitive 4D spectra in a reasonable time. Triple sensitivity-enhanced 4D hCACONH and hCOCANH were recorded in ca. 48 h each, hCXCANH in ca. 100 h and hCXCAcoNH in ca. 170 h. All 4D spectra were recorded at 55 kHz MAS on a 600 MHz spectrometer, using 5% NUS. The experiments for chemical shift assignments were performed on a back-exchanged perdeuterated sample. This makes experiments for side-chain assignments challenging since the pool of initial magnetization in the ^1^H- ^13^C CP step is limited to back-exchanged sites, mostly amide protons. With this in mind the main goal was to obtain Cβ assignments. Unambiguous assignments of GlpG would not be possible without access to at least Cβ chemical shifts, additional side-chain information is of course highly beneficial. The initial ^1^H- ^13^C CP was optimized for highest intensity in the region around 10–40 ppm, where most Cβ and other expected side-chains appear. Longer mixing time could potentially improve the side-chain assignments of carbon atoms further down the side-chain, but will lead to an overall loss in sensitivity. Figure 3B shows an example of side-chain assignments for residues W38-Q44 based on the sensitivity-enhanced 4D TOCSY experiments, Fig. 3A shows how the spectra are connected in the strip plots. All the Cβ chemical shifts of residues W38-Q44 could be assigned and, additionally, the Cγ chemical shifts of E42 and Q44 were also assigned. But for the leucine residue (L39), with a hydrophobic branched side-chain, only the Cβ was visible in the spectra. Figure 3C shows an AlphaFold2 (Jumper et al. 2021; Varadi et al. 2024) model of GlpG with the residues coloured depending on the assignments (see SI Tables 4, 5 and 6 for assigned chemical shifts). In total, utilizing sensitivity-enhanced 3D and 4D spectra for backbone and side-chain assignments, the Cα chemical shifts of 133 residues were successfully assigned (137 residues with any atom assigned). Among these, fifteen residues are glycines and prolines, leaving 118 residues for potential side-chain assignments. Of these, the Cβ chemical shifts were assigned in 91 residues (green in Fig. 3C), while in 7 residues where the Cβ could not be assigned, another side-chain was assigned (orange in Fig. 3C). Consequently, only 20 residues remain without any side-chain assignments (dark gray in Fig. 3C). Figure 3D provides a comprehensive analysis of side-chain assignments for each residue type in GlpG, excluding glycine and proline. Side-chains that were consistently assigned are denoted in green, those occasionally assigned in orange, and those not assigned in red. This pattern aligns relatively well with expected outcomes for side-chain assignments in a perdeuterated sample, where Cβ chemical shifts are typically available to be assigned, and carbons further along the side-chain are less frequently assignable. Notable exceptions where Cβ was not consistently assigned include glutamates (3/8), threonines (2/6), and valines (1/10). For glutamates, the missing assignments appear to be due to the residues’ positioning within the protein. For example, two of the glutamates lacking Cβ assignments (E118 and E166) are residues where the amide protons could not be assigned, but the Cα (and CO) chemical shifts were determined using the amide protons of the preceding residues in the 4D hCACONH spectrum. The reason for the lack of Cβ assignments of several threonines remains unclear, especially since the Cγ were assigned in 5 out of 6 threonines. The sole threonine with a missing Cγ assignment is T97, which is similarly situated as E118 and E166.

A plausible explanation for the missing threonine Cβ assignments could be unfavorable dynamics, resulting in inadequate coherence lifetimes for the many magnetization transfer steps in the 4D TOCSY experiments, while the fast-moving methyl groups (Cγ) have longer coherence lifetimes, thus detectable in TOCSY experiments. Another possible reason is the close similarity between Cα and Cβ chemical shifts in some threonines, hindering their distinction. Valines, expected to behave similarly to other residues with branched hydrophobic side-chains such as leucines and isoleucines, showed an unexpected pattern, with only 1 out of 10 valine Cβ assigned. There is no apparent explanation for this discrepancy. Future structural and dynamic studies of GlpG may elucidate why the valine side-chains are challenging to assign. Intriguingly, among the side-chains within the TOCSY mixing range, the only ones never appearing in the spectra are the Cγ and Cδ of isoleucines and leucines, and the Cε of lysines (see Fig. 3D). This implies that nearly complete side-chain assignments are achievable using sensitivity-enhanced TOCSY experiments in solid-state NMR of perdeuterated proteins at 55 kHz. Given the complexity of GlpG as a membrane protein, numerous factors could contribute to the difficulty in assigning certain residues, such as unfavourable dynamics, lipid interactions, incomplete back-exchange of exchangeable protons, and overlapping peaks.

Leveraging the high performance of the newly developed hSPEPS for dipolar-based ^13^Cβ- ^13^Cα transfers, we assessed whether the extended mixing time and reliance on J-coupling transfers in TOCSY could explain some missing Cβ assignments. We recorded double sensitivity-enhanced 3D hCXcaNH spectra employing hSPEPS and zTOCSY. However, no additional Cβ peaks, unassigned in the 4D TOCSY spectra, were identified in the 3D hSPEPS spectrum. A comparative analysis of the intensities of isolated Cβ-N-H peaks in the 3D spectra indicated that zTOCSY generally outperformed hSPEPS (see SI Fig. 11). This suggests that the TOCSY-based approach is suitable for membrane proteins, and the missing side-chain assignments in GlpG are likely attributed to internal dynamics and lipid-protein interactions interfering with both dipolar and J-coupling-based magnetization transfers.

TOCSY mixing is highly efficient for fully protonated proteins at MAS rates of 100 kHz and above (Stanek et al. 2016). Due to the increased efficiency of the 2022^1^H- ^13^C magnetization transfer step that can be achieved in fully protonated proteins compared to in H_2_O back-exchanged perdeuterated proteins, we expect that the described approach will be even more advantageous when applied to fully protonated samples at faster MAS rates.

## Concluding remarks

This study demonstrates the efficiency of ^13^C−^13^C TOCSY mixing via the DIPSI-3 mixing sequence for sensitivity-enhanced solid-state NMR experiments. 90 degree pulses before and after the DIPSI-3 mixing sequence are important to prevent unwanted cross-transfers resulting in mirrored peaks and loss of sensitivity. The methodology can be seamlessly integrated with advanced sensitivity-enhanced mixing sequences, such as TROP and SPEPS, which facilitate transfers between backbone atoms. Consequently, it enables the construction of sensitivity-enhanced 4D experiments, thereby facilitating the side-chain assignments of complex membrane proteins. We validated this approach using a back-exchanged perdeuterated protein sample of the full length 30 kDa membrane protein GlpG. A comprehensive dataset, suitable for backbone and side-chain assignments, comprising two double sensitivity-enhanced 3D spectra and four triple sensitivity-enhanced 4D spectra was acquired within a three-week period at 55 kHz MAS on a 600 MHz spectrometer. Interestingly, despite utilizing a back-exchanged perdeuterated sample, all anticipated side-chain carbons for all residue types, excluding the Cγ and Cδ of isoleucines and leucines and the Cε of lysines, were detectable. This approach should prove even more advantageous for proteins with higher degrees of side-chain protonation that enables more efficient ^1^H- ^13^C magnetization transfers compared to back-exchanged perdeuterated samples, provided that sufficiently fast MAS rates and/or high external magnetic field strengths are applied.

## Electronic supplementary material

Below is the link to the electronic supplementary material.

Supplementary Material 1

## Data Availability

The chemical shift assignments of GlpG are available in the supplementary information and deposited to the BMRB under accession number 52584. All information needed to reproduce the experiments in this study is included in the article and supplementary information. Further inquiries are available from the corresponding authors upon reasonable request.
