# Supplementary material for: Evaluation of TOCSY mixing for sensitivity-enhancement in solid-state NMR and application of 4D experiments for side-chain assignments of the full-length 30 kDa membrane protein GlpG
Source: J Biomol NMR. 2025 Jan 22;79(1):25–34. doi: 10.1007/s10858-024-00454-7 (PMC11832555; doi:10.1007/s10858-024-00454-7)
Supplement: Supplementary file 1 — Supplementary Material 1 [file 10858_2024_454_MOESM1_ESM.docx]

**Supplementary information for**

Evaluation of TOCSY mixing for sensitivity-enhancement in solid-state NMR and application of 4D experiments for side-chain assignments of the full-length 30 kDa membrane protein GlpG

**Author information**

Carl Öster^‡^*
ORCID: 0000-0002-8723-4533
email: oester@fmp-berlin.de

Veniamin Chevelkov^‡^
ORCID: 0000-0002-7615-3535

Adam Lange^‡§^*
ORCID: 0000-0002-7534-5973
email: alange@fmp-berlin.de

^‡^Research Unit Molecular Biophysics, Leibniz-Forschungsinstitut für Molekulare Pharmakologie, Robert-Rössle-Straße 10, 13125 Berlin, Germany

^§^Institut für Biologie, Humboldt-Universität zu Berlin, Invalidenstraße 42, 10115 Berlin, Germany

*Corresponding authors

**Abstract**

Chemical shift assignments of large membrane proteins by solid-state NMR experiments are challenging. Recent advancements in sensitivity-enhanced pulse sequences, have made it feasible to acquire ^1^H-detected 4D spectra of these challenging protein samples within reasonable timeframes. However, obtaining unambiguous assignments remains difficult without access to side-chain chemical shifts. Drawing inspiration from sensitivity-enhanced TOCSY experiments in solution NMR, we have explored the potential of ^13^C-^13^C TOCSY mixing as a viable option for triple sensitivity-enhanced 4D experiments aimed at side-chain assignments in solid-state NMR. Through simulations and experimental trials, we have identified optimal conditions to achieve uniform transfer efficiency for both transverse components and to minimize undesired cross-transfers. Our experiments, conducted on the 30 kDa membrane protein GlpG embedded in *E. coli* liposomes, have demonstrated enhanced sensitivity compared to the most effective dipolar and J-coupling-based ^13^C-^13^C mixing sequences. Notably, a non-uniformly sampled 4D hCXCANH spectrum with exceptionally high sensitivity was obtained in just a few days using a 600 MHz spectrometer equipped with a 1.3 mm probe operating at a magic angle spinning rate of 55 kHz.

**Supplementary text**

Selected sections of the SIMPSON^1^ input file, which represent relevant parameters for the recoupling sequences.

spinsys {

channels 13C

nuclei 13C 13C

jcoupling 1 2 35 0 0 0 0 0

shift 1 15p -60p 0.89 17 84 12

shift 2 -15p -13p 0.29 77 54 22

dipole 1 2 -2084.9 0 93 -94

}

par {

spin_rate 55000

gamma_angles 12

variable NdipsiP 250

sw spin_rate/NdipsiP

crystal_file rep256

start_operator I1z

detect_operator I2z

proton_frequency 600e6

}

**Tables**

Table S 1. Parameters for all magnetization transfer steps in the sensitivity-enhanced 3D and 4D experiments used for assignments of GlpG. The nutation frequency is given for the maximum amplitude of each pulse.

| Experiment | hCANH | hCONH | hCACONH | hCOCANH | hCXCANH | hCXCAcoNH |
| --- | --- | --- | --- | --- | --- | --- |
| **^1^H-^13^C** |  |  |  |  |  |  |
| ^1^H shape | ramp 90-100 | ramp 90-100 | ramp 90-100 | ramp 90-100 | ramp 90-100 | ramp 90-100 |
| ^1^H power (kHz) | 82 | 82 | 82 | 82 | 80 | 80 |
| ^13^C shape | rectangular | rectangular | rectangular | rectangular | rectangular | rectangular |
| ^13^C power (kHz) | 20 | 20 | 20 | 20 | 20 | 20 |
| duration (ms) | 1.8 | 1.8 | 1.8 | 1.8 | 8 | 8 |
| **^13^C-^13^C (side-chains)** |  |  |  |  |  |  |
| ^13^C shape |  |  |  |  | DIPSI 3 | DIPSI 3 |
| ^13^C power (kHz) |  |  |  |  | 15.625 | 15.625 |
| duration (ms) |  |  |  |  | 13.9 | 13.9 |
| **^13^C-^13^C (backbone)** |  |  |  |  |  |  |
| ^13^C shape | - | - | homo TROP | homo TROP |  | homo TROP |
| ^13^C power (kHz) | - | - | 73 | 73 |  | 73 |
| duration (ms) | - | - | 1.8 | 1.8 |  | 1.8 |
| **^13^C-^15^N** |  |  |  |  |  |  |
| ^13^C shape | TROP | TROP | TROP | TROP | TROP | TROP |
| ^13^C power (kHz) | 64 | 66 | 66 | 64 | 67 | 67 |
| ^15^N shape | TROP | TROP | TROP | TROP | TROP | TROP |
| ^15^N power (kHz) | 42 | 42 | 42 | 42 | 42 | 42 |
| duration (ms) | 3.64 | 3.64 | 3.64 | 3.64 | 3.64 | 3.64 |
| **^15^N-^1^H** |  |  |  |  |  |  |
| ^15^N shape | TROP | TROP | TROP | TROP | TROP | TROP |
| ^15^N power (kHz) | 55 | 55 | 55 | 55 | 55 | 55 |
| ^1^H shape | TROP | TROP | TROP | TROP | TROP | TROP |
| ^1^H power (kHz) | 68 | 68 | 68 | 68 | 63 | 63 |
| duration (ms) | 0.8 | 0.8 | 0.8 | 0.8 | 0.8 | 0.8 |

Table S 2. Acquisition parameters for all sensitivity-enhanced 3D and 4D experiments used for the assignments of GlpG. SW = spectral width, NUS = Non-uniform sampling.

| Experiment | hCANH | hCONH | hCACONH | hCOCANH | hCXCANH | hCXCAcoNH |
| --- | --- | --- | --- | --- | --- | --- |
| Scans | 64 | 48 | 48 | 48 | 96 | 152 |
| ^1^H acquisition time (ms) | 30 | 30 | 30 | 30 | 21.3 | 21.3 |
| ^1^H points | 1438 | 1438 | 1438 | 1438 | 1024 | 1024 |
| ^1^H SW (ppm) | 40 | 40 | 40 | 40 | 40 | 40 |
| ^13^CA acquisition time (ms) | 5.1 | - | 4.4 | 4.4 | 3.5 | 3.5 |
| ^13^CA points | 46 | - | 40 | 40 | 32 | 32 |
| ^13^C SW (ppm) | 30 | - | 30 | 30 | 30 | 30 |
| ^13^CO acquisition time (ms) | - | 8.2 | 8.2 | 8.2 | - | - |
| ^13^CO points | - | 32 | 32 | 32 | - | - |
| ^13^CO SW (ppm) | - | 13 | 13 | 13 | - | - |
| ^13^CX acquisition time (ms) | - | - | - | - | 3.4 | 3.4 |
| ^13^CX points | - | - | - | - | 64 | 64 |
| ^13^CX SW | - | - | - | - | 63 | 63 |
| ^15^N acquisition time (ms) | 14.3 | 14.7 | 11.3 | 11.3 | 8.5 | 9.1 |
| ^15^N points | 54 | 52 | 40 | 40 | 32 | 32 |
| ^15^N SW (ms) | 31 | 29 | 29 | 29 | 31 | 29 |
| NUS points | - | - | 321 | 322 | 411 | 411 |
| NUS % | - | - | 5 | 5 | 5 | 5 |
| total points | 2484 | 1664 | 2568/51200 | 2576/51200 | 3288/81920 | 3288/81920 |
| Experimental time (h) | 65.5 | 40 | 47 | 49 | 103.5 | 170 |

Table S 3. NmrPipe^2^ processing parameters for reconstruction of 4D NUS spectra recorded on GlpG. The spectra were reconstructed using the ist4D.com script in nmrPipe. The ssNMR flag was used for all spectra.

| Parameter | hCACONH | hCOCANH | hCXCANH | hCXCAcoNH |
| --- | --- | --- | --- | --- |
| istMaxRes | 18 | 18 | 7 | 9 |
| **x dimension** | x = ^1^H | x = ^1^H | x = ^1^H | x = ^1^H |
| xAPOD | GM | GM | GM | GM |
| xGLB | 60 | 60 | 60 | 60 |
| xEXTX1 | 11.3ppm | 11.3ppm | 11.3ppm | 11.3ppm |
| xEXTXN | 5.7ppm | 5.3ppm | 5.53ppm | 6ppm |
| xP0 | -114 | -126 | -112 | -112 |
| **y dimension** | y = ^15^N | y = ^15^N | y = ^15^N | y = ^15^N |
| yNUSZF | None | None | size=22 | size=22 |
| yZFARG | size=128 | size=128 | size=96 | size=96 |
| yFTARG | neg | neg | neg | neg |
| yP0 | 90 | 90 | 90 | 90 |
| **z dimension** | z = ^13^CO | z = ^13^CA | z = ^13^CA | z = ^13^CA |
| zNUSZF | None | None | size=22 | size=22 |
| zZFARG | size=64 | size=128 | size=96 | size=96 |
| zFTARG | neg | neg | neg | neg |
| zP0 | 90 | 90 | 90 | 90 |
| **a dimension** | a = ^13^CA | a = ^13^CO | a = ^13^CX | a = ^13^CX |
| aNUSZF | None | None | size=42 | size=42 |
| aZFARG | size=128 | size=64 | size=256 | size=256 |
| aFTARG | - | neg | neg | neg |
| aP0 | 0 | 0 | 0 | 0 |

Table S 4. Assignment table for 100% H_2_O back-exchanged ^2^H^13^C^15^N labelled GlpG (part 1/3). Chemical shifts were referenced using internal DSS and are deposited in the BMRB (ID: 52584)

| Number | Type | H | N | C | Cα | Cβ | Cγ | Cδ |
| --- | --- | --- | --- | --- | --- | --- | --- | --- |
| 2 | LEU | 8.85 | 121.75 | 174.92 | 53.55 | 44.84 |  |  |
| 3 | MET | 8.76 | 128.95 | 175.78 | 55.50 | 31.03 |  |  |
| 4 | ILE | 8.89 | 127.07 | 175.21 | 59.48 | 35.81 |  |  |
| 5 | THR | 7.26 | 109.18 | 170.31 | 59.85 | 67.59 | 17.22 |  |
| 6 | SER | 7.74 | 115.60 | 173.06 | 56.54 | 64.63 |  |  |
| 7 | PHE | 8.97 | 121.81 | 175.28 | 56.36 | 42.59 |  |  |
| 8 | ALA | 8.93 | 125.03 | 177.57 | 53.33 | 17.82 |  |  |
| 9 | ASN | 8.22 | 116.99 |  | 49.43 | 39.15 |  |  |
| 10 | PRO |  |  | 179.42 |  |  |  |  |
| 11 | ARG | 8.11 | 118.56 | 179.56 | 58.23 | 28.49 |  |  |
| 12 | VAL | 7.24 | 122.81 | 176.90 | 65.23 |  | 20.81 |  |
| 13 | ALA | 6.77 | 118.63 | 178.61 | 54.12 | 17.62 |  |  |
| 14 | GLN | 7.79 | 114.56 | 176.80 | 57.09 | 27.75 | 32.61 |  |
| 15 | ALA | 7.62 | 121.36 | 180.11 | 54.75 | 16.68 |  |  |
| 16 | PHE | 7.49 | 118.93 | 177.06 | 61.16 | 37.55 |  |  |
| 17 | VAL | 8.03 | 119.35 | 179.19 | 66.96 |  |  |  |
| 18 | ASP | 9.38 | 122.81 | 179.53 | 56.95 | 38.82 |  |  |
| 19 | TYR | 8.37 | 120.70 | 179.73 | 62.10 | 37.46 |  |  |
| 20 | MET | 8.54 | 119.57 | 179.28 | 55.96 | 26.85 | 30.95 |  |
| 21 | ALA | 8.09 | 123.12 | 182.12 | 55.15 | 16.54 |  |  |
| 22 | THR | 7.52 | 110.57 | 175.14 | 64.23 |  | 20.53 |  |
| 23 | GLN | 7.16 | 117.76 | 175.80 | 53.05 |  | 32.18 |  |
| 24 | GLY | 7.67 | 106.19 | 173.59 | 45.18 |  |  |  |
| 25 | VAL | 7.74 | 120.42 | 174.28 | 60.42 |  |  |  |
| 26 | ILE | 9.06 | 128.51 | 175.14 | 59.73 | 35.67 |  |  |
| 27 | LEU | 8.29 | 129.42 | 175.07 | 52.60 | 43.57 |  |  |
| 28 | THR | 9.03 | 112.80 | 173.44 | 60.34 |  | 20.49 |  |
| 29 | ILE | 8.80 | 122.77 | 176.77 | 59.76 | 39.75 |  |  |
| 30 | GLN | 9.50 | 127.69 | 174.53 | 54.36 | 30.68 | 32.96 |  |
| 31 | GLN | 9.10 | 126.34 |  | 54.69 |  |  |  |
| 35 | SER | 8.97 | 120.41 | 172.72 | 57.34 | 63.86 |  |  |
| 36 | ASP | 8.89 | 127.39 | 175.01 | 53.52 | 41.33 |  |  |
| 37 | VAL | 8.65 | 122.07 | 174.14 | 61.43 |  |  |  |
| 38 | TRP | 9.64 | 128.22 | 174.37 | 56.17 | 31.84 |  |  |
| 39 | LEU | 9.56 | 122.67 | 175.22 | 53.12 | 45.89 |  |  |
| 40 | ALA | 8.73 | 128.41 | 177.14 | 53.26 | 18.49 |  |  |
| 41 | ASP | 7.37 | 115.16 | 176.09 | 51.76 | 39.17 |  |  |
| 42 | GLU | 8.85 | 124.04 | 178.15 | 58.90 | 28.08 | 35.32 |  |
| 43 | SER | 8.46 | 116.88 | 176.27 | 61.05 | 62.39 |  |  |
| 44 | GLN | 7.83 | 118.75 | 176.05 | 54.29 | 25.49 | 32.14 |  |
| 45 | ALA | 7.62 | 122.51 | 178.61 | 56.43 | 17.61 |  |  |
| 46 | GLU | 8.73 | 116.95 | 179.54 | 59.41 | 27.90 | 35.69 |  |
| 47 | ARG | 8.15 | 121.73 | 179.09 | 58.52 | 28.88 | 25.16 |  |
| 48 | VAL | 8.50 | 118.47 | 177.99 | 66.55 |  |  |  |
| 49 | ARG | 8.78 | 119.25 | 179.25 | 60.47 | 28.35 |  |  |
| 50 | ALA | 8.18 | 123.16 | 181.41 | 54.93 | 16.73 |  |  |

Table S 5. Assignment table for GlpG (part 2/3).

| Number | Type | H | N | C | Cα | Cβ | Cγ | Cδ |
| --- | --- | --- | --- | --- | --- | --- | --- | --- |
| 51 | GLU | 8.43 | 118.32 | 180.38 | 57.93 |  |  |  |
| 52 | LEU | 9.28 | 124.01 | 177.17 | 57.36 | 40.13 |  |  |
| 53 | ALA | 7.60 | 119.37 | 180.35 | 54.83 | 16.73 |  |  |
| 54 | ARG | 7.27 | 116.55 | 178.34 | 58.56 |  |  |  |
| 55 | PHE | 8.50 | 123.53 | 176.55 | 60.07 | 38.90 |  |  |
| 56 | LEU | 8.48 | 114.76 | 179.77 | 56.13 | 40.85 |  |  |
| 57 | GLU | 7.33 | 117.08 | 176.35 | 58.58 |  |  |  |
| 95 | PRO |  |  | 178.91 | 65.54 |  |  |  |
| 96 | VAL | 10.82 | 121.99 | 178.34 | 66.93 |  | 23.19 |  |
| 97 | THR |  |  | 179.52 | 67.22 |  |  |  |
| 98 | TRP | 8.37 | 119.47 | 178.78 | 60.94 | 30.33 |  |  |
| 107 | VAL | 8.26 | 118.44 | 177.99 | 66.36 |  |  |  |
| 108 | PHE | 8.81 | 119.27 | 178.72 | 56.31 | 39.20 |  |  |
| 113 | ILE |  |  | 177.07 | 63.59 |  |  |  |
| 114 | LEU | 8.87 | 116.48 | 178.19 | 55.11 | 41.98 |  |  |
| 115 | GLY | 7.19 | 108.72 | 174.07 | 44.11 |  |  |  |
| 116 | ASP | 8.08 | 120.23 | 178.60 | 57.63 | 40.04 |  |  |
| 118 | GLU |  |  | 177.70 | 58.86 |  |  |  |
| 119 | VAL | 6.94 | 115.01 | 178.60 | 65.79 |  |  |  |
| 120 | MET | 8.74 | 120.26 | 177.77 | 59.26 | 31.79 |  |  |
| 121 | LEU | 7.95 | 115.70 | 179.38 | 57.87 | 40.32 |  |  |
| 122 | TRP | 7.37 | 117.06 | 177.76 | 58.32 |  |  |  |
| 123 | LEU |  |  | 177.37 | 54.36 | 42.18 |  |  |
| 124 | ALA | 8.77 | 118.86 | 176.07 | 52.61 | 19.28 |  |  |
| 125 | TRP | 8.08 | 119.08 |  | 55.24 | 28.66 |  |  |
| 126 | PRO |  |  | 177.53 |  |  |  |  |
| 127 | PHE | 5.77 | 117.97 | 173.26 | 53.76 | 36.38 |  |  |
| 128 | ASP | 7.09 | 121.37 | 174.72 | 51.82 | 47.19 |  |  |
| 129 | PRO |  |  | 178.53 |  |  |  |  |
| 130 | THR | 8.66 | 108.91 | 176.26 | 63.93 | 64.38 | 20.84 |  |
| 131 | LEU | 7.74 | 119.71 | 177.66 | 53.65 | 40.20 |  |  |
| 132 | LYS | 6.89 | 115.75 | 176.38 | 58.05 | 30.98 |  |  |
| 133 | PHE | 8.48 | 115.24 | 175.13 | 56.33 | 36.54 |  |  |
| 134 | GLU | 7.18 | 120.26 | 176.97 | 54.69 |  | 35.08 |  |
| 135 | PHE | 7.55 | 116.83 | 175.42 | 59.77 | 39.33 |  |  |
| 136 | TRP | 6.84 | 115.68 | 176.68 | 60.30 | 26.29 |  |  |
| 137 | ARG | 7.03 | 122.00 | 175.52 | 57.13 | 28.30 |  | 41.75 |
| 138 | TYR | 6.41 | 111.57 | 173.96 | 59.22 | 35.46 |  |  |
| 139 | PHE | 8.28 | 113.05 | 176.60 | 58.21 | 41.72 |  |  |
| 140 | THR | 9.24 | 107.28 | 175.18 | 65.76 |  | 21.26 |  |
| 141 | HIS | 7.11 | 121.11 | 177.41 | 56.87 | 29.50 |  |  |
| 142 | ALA | 6.74 | 123.06 | 174.91 | 52.17 | 16.33 |  |  |
| 143 | LEU | 7.26 | 107.48 | 177.55 | 53.57 | 41.64 |  |  |
| 144 | MET | 6.78 | 115.74 | 171.70 | 56.03 | 30.88 |  |  |
| 145 | HIS | 6.34 | 112.32 | 175.44 | 55.93 | 32.75 |  |  |

Table S 6. Assignment table for GlpG (part 3/3).

| Number | Type | H | N | CO | Cα | Cβ | Cγ | Cδ |
| --- | --- | --- | --- | --- | --- | --- | --- | --- |
| 146 | PHE | 10.57 | 118.27 | 175.30 | 59.46 | 39.73 |  |  |
| 147 | SER | 8.14 | 112.01 | 174.01 | 56.92 | 65.79 |  |  |
| 148 | LEU | 9.87 | 123.96 | 177.98 | 57.02 | 40.58 |  |  |
| 149 | MET | 8.90 | 116.21 | 177.35 | 58.07 | 31.55 |  |  |
| 162 | GLY |  |  | 175.54 | 46.13 |  |  |  |
| 163 | GLY | 6.87 | 106.35 | 176.06 | 46.76 |  |  |  |
| 164 | ALA | 6.65 | 121.78 | 179.81 | 54.67 | 16.69 |  |  |
| 166 | GLU |  |  | 179.27 | 58.70 |  |  |  |
| 167 | LYS | 8.34 | 116.76 | 178.40 | 57.79 | 30.87 | 23.36 |  |
| 168 | ARG | 7.87 | 113.80 | 177.55 | 56.18 | 29.53 | 25.70 | 41.77 |
| 169 | LEU | 8.87 | 116.51 | 177.64 | 53.88 | 41.63 |  |  |
| 170 | GLY | 7.56 | 110.14 | 173.95 | 44.60 |  |  |  |
| 171 | SER | 8.77 | 116.41 | 175.94 | 62.90 | 63.30 |  |  |
| 172 | GLY | 9.25 | 109.26 | 174.83 | 47.23 |  |  |  |
| 173 | LYS | 8.06 | 121.39 | 177.77 | 56.36 | 27.67 | 21.61 | 24.63 |
| 174 | LEU | 7.08 | 115.85 | 179.99 | 57.16 | 40.15 |  |  |
| 175 | ILE | 8.65 | 121.46 |  | 65.95 | 36.58 |  |  |
| 188 | VAL |  |  | 177.04 | 66.14 | 28.39 |  |  |
| 189 | GLN | 7.88 | 119.21 | 178.08 | 58.01 | 25.29 |  |  |
| 190 | GLN | 7.63 | 116.29 | 178.32 | 56.50 | 23.61 | 31.00 |  |
| 193 | SER |  |  |  | 58.45 | 64.96 |  |  |
| 194 | GLY | 7.98 | 111.43 |  | 44.65 |  |  |  |
| 197 | PHE |  |  | 172.97 | 55.90 | 39.31 |  |  |
| 198 | GLY | 8.50 | 104.18 | 173.70 | 46.61 |  |  |  |
| 199 | GLY | 8.34 | 109.98 | 177.74 | 44.97 |  |  |  |
| 200 | LEU |  |  | 178.37 | 54.97 |  |  |  |
| 201 | SER | 8.31 | 115.36 | 176.21 | 63.43 |  |  |  |
| 202 | GLY | 8.89 | 111.28 | 175.07 | 47.21 |  |  |  |
| 203 | VAL | 7.78 | 122.71 |  | 64.17 |  |  |  |
| 212 | TRP |  |  | 178.28 |  |  |  |  |
| 213 | LEU | 8.28 | 117.52 | 178.77 | 57.14 | 39.05 |  |  |
| 214 | ARG | 8.73 | 119.19 | 178.02 | 58.09 | 26.91 | 24.47 | 41.92 |
| 215 | GLY | 7.67 | 104.51 | 176.15 | 47.87 |  |  |  |
| 216 | GLU | 7.30 | 117.79 | 178.68 | 57.38 | 28.62 | 33.62 |  |
| 217 | ARG | 8.08 | 114.34 | 176.27 | 56.39 | 30.18 | 26.21 |  |
| 218 | ASP | 8.37 | 117.51 | 173.45 | 51.15 | 41.35 |  |  |
| 219 | PRO |  |  | 179.52 | 64.82 |  |  |  |
| 220 | GLN | 8.81 | 117.84 | 177.02 | 57.26 | 26.91 | 33.41 |  |
| 221 | SER | 8.15 | 115.61 | 175.98 | 60.76 | 63.48 |  |  |
| 222 | GLY | 7.75 | 106.88 | 173.34 | 45.38 |  |  |  |
| 223 | ILE |  |  | 173.50 | 58.50 | 38.91 |  |  |
| 224 | TYR | 7.62 | 118.82 | 173.17 | 56.73 | 37.73 |  |  |
| 225 | LEU | 8.98 | 124.61 | 174.57 | 52.86 |  |  |  |
| 255 | ILE | 7.79 | 116.94 | 176.94 | 63.48 |  |  |  |
| 256 | ALA | 7.98 | 121.16 | 178.85 | 54.94 | 16.78 |  |  |
| 257 | GLY | 8.00 | 102.70 |  | 47.26 |  |  |  |

**Figures**

**
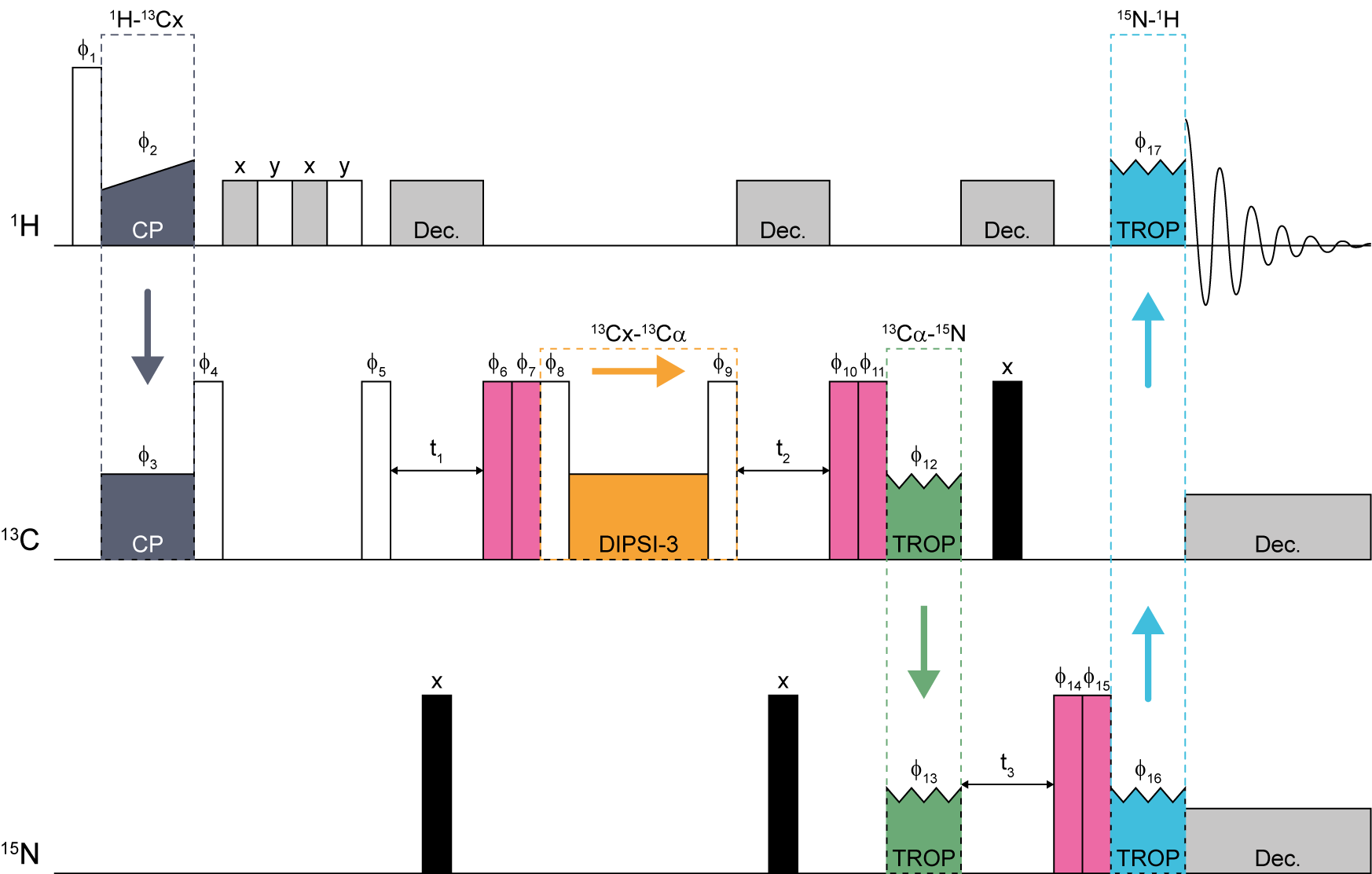
**

Figure S 1. Pulse sequence for the triple sensitivity-enhanced 4D hCXCANH experiment, modified from the double sensitivity-enhanced hCANH^3^ downloaded from [www.optimal-nmr.net](http://www.optimal-nmr.net). The hetero- and homo-nuclear magnetization transfer steps are indicated in dark grey (^1^H-^13^Cx, ^13^C offset at 42.2 ppm), orange (^13^Cx-^13^Cα), green (^13^Cα-^15^N, ^13^C offset at 56.2 ppm) and blue (^15^N-^1^H). 90 degree pulses are shown as white and pink bars, the pink bars are the pulses used for Echo-Antiecho determination. 180 degree pulses are shown as black bars. The ^13^C offset was set in the middle between the ^13^Cα and ^13^CO chemical shift regions (at 120 ppm) for the 180 degree pulse on the ^13^C channel during ^15^N indirect acquisition. Low power decoupling periods (10 kHz Waltz-64^4^) are shown as light grey blocks and MISSISSIPPI^5^ water suppression is shown as an alternating white and light grey block. The phases are as follows: φ_1_ = 1, φ_2_ = 0, φ_3_= 1 3, φ_4_ = 0 2, φ_5_ = 2 0, φ_6_ = 0, φ_7_ = 2, φ_8_ = 0, φ_9_ = 2, φ_10_= 0, φ_11_= 2, φ_12_= 0, φ_13_ = 0 0 2 2, φ_14_ = 0, φ_15_ = 2, φ_16_ 0, φ_17_ = 0, φ_acquisition_ = 1 3 3 1, with 0 corresponding to x, 1 to y, 2 to -x and 3 to -y. Echo-Antiecho was used for all indirect dimensions. The phase and time increments were performed as follows (in a Bruker pulse program) :
F1EA(calph(ph7, +180),caldel(d0, +in0)) ; ^13^Cx dimension
F2EA(calph(ph11, +180) & calph(ph7, +180),caldel(d11, +in11)) ; ^13^Cα dimension
F3EA(calph(ph15, +180) & calph(ph11, +180),caldel(d10, +in10)) ; ^15^N dimension
where ph7 = φ_7_, ph11 = φ_11_, ph15 = φ_15_, d0 and in0 corresponds to t_1_, d11 and in11 corresponds to t_2_, and d10 and in10 corresponds to t_3_. For the 4D hCXCAcoNH pulse sequence a homoTROP CA-CO pulse^6^ was added after the indirect ^13^Cα acquisition and ^13^CO-^15^N TROP transfer (with the ^13^C offset at 176.2 ppm) was used instead of the ^13^Cα-^15^N transfer.


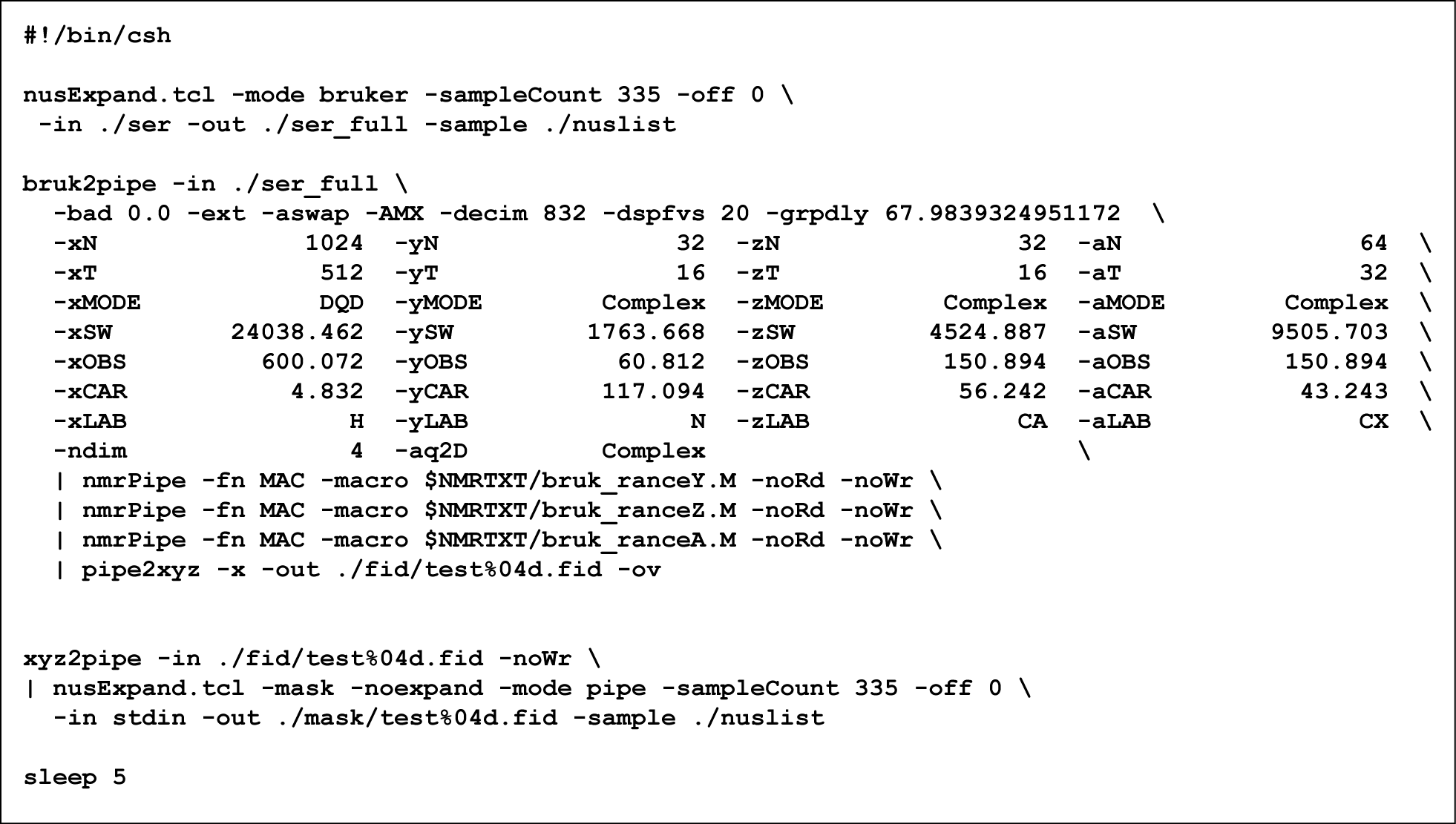


Figure S 2. Example of nmrPipe^2^ script for conversion of the raw data of a triple sensitivity-enhanced 4D experiment (hCXCAcoNH) from Bruker to nmrPipe format.


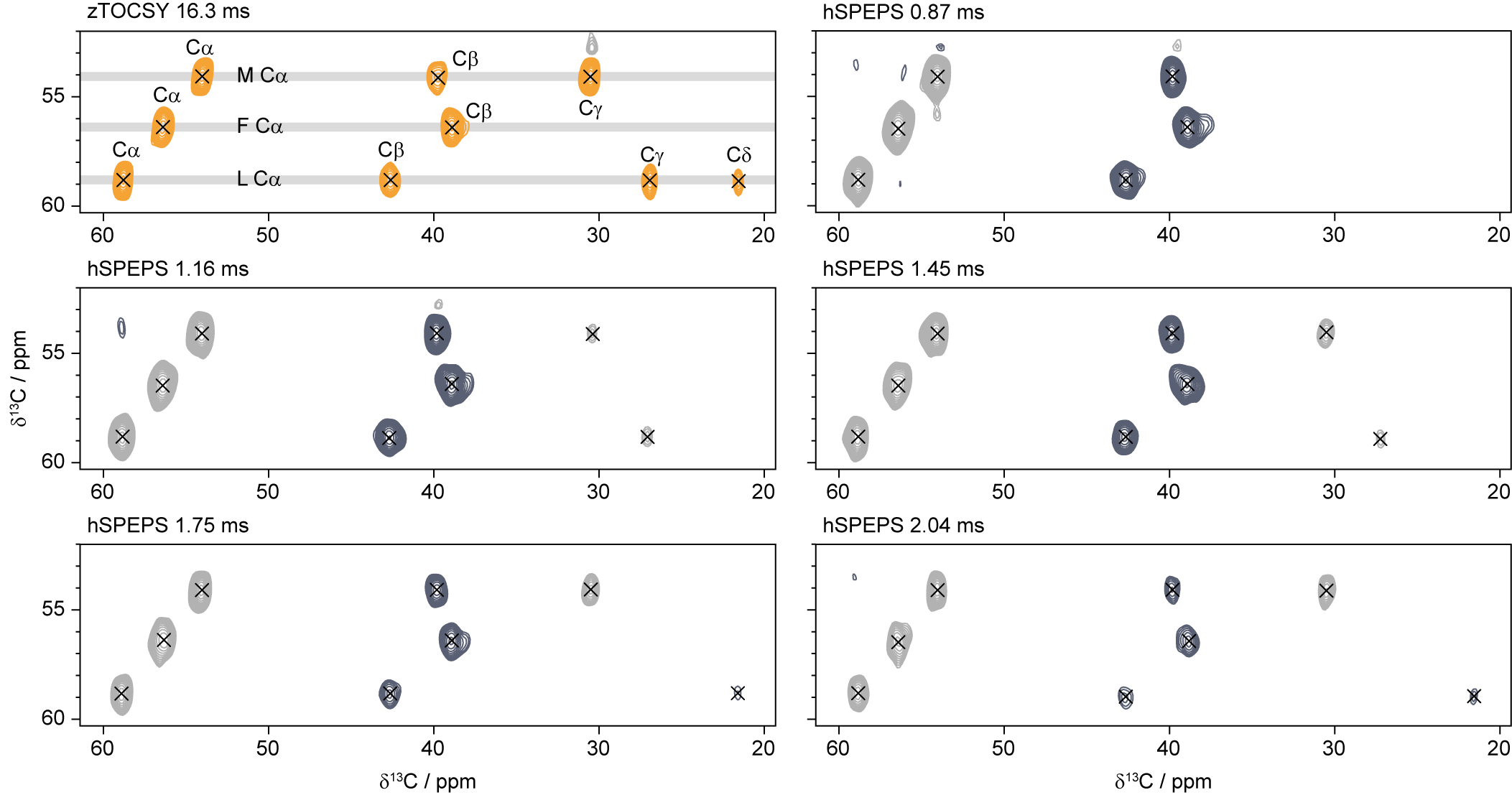


Figure S 3. Comparison of sensitivity-enhanced hnCACX spectra recorded on MLF using hSPEPS^7^ (for ^13^Cα-^13^Cx transfers) with different mixing times. 0.87 ms mixing gave the highest intensity for the Cα-Cβ cross-peaks and was therefore used in the analysis in Fig.1 in the main article. Longer mixing times resulted in transfers to peaks further down the side-chains, as expected^7^. A spectrum recorded using zTOCSY (orange) with the chemical shift assignments of MLF indicated is shown as a comparison.


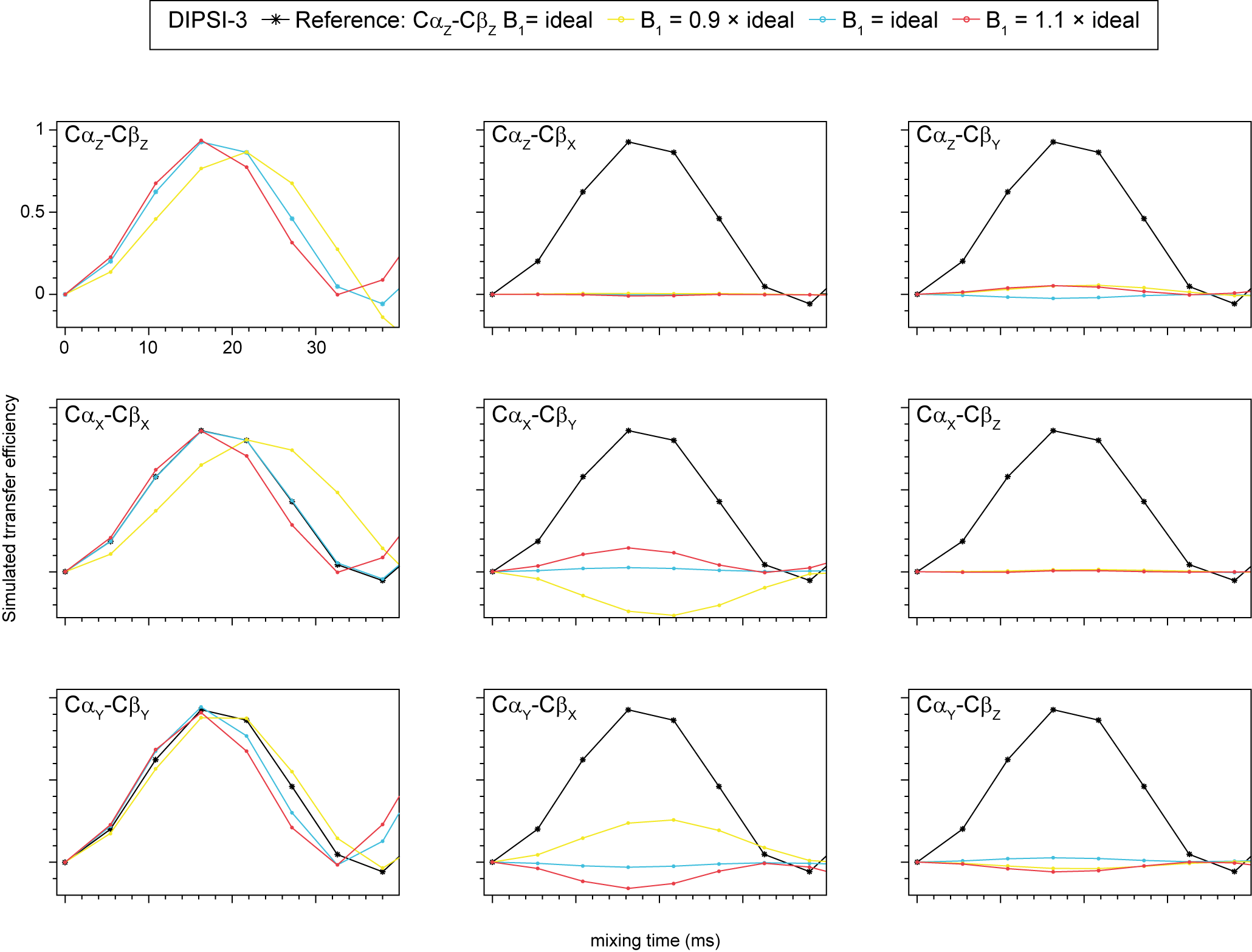


Figure S 4. Simulations of polarization transfer dynamics between different components of two ^13^C spins representing Cα and Cβ nuclei for DIPSI-3 mixing. The nutation frequency was set to 10 kHz (blue lines). The effect of radio-frequency inhomogeneity was simulated by using non-ideal conditions (yellow and red lines).


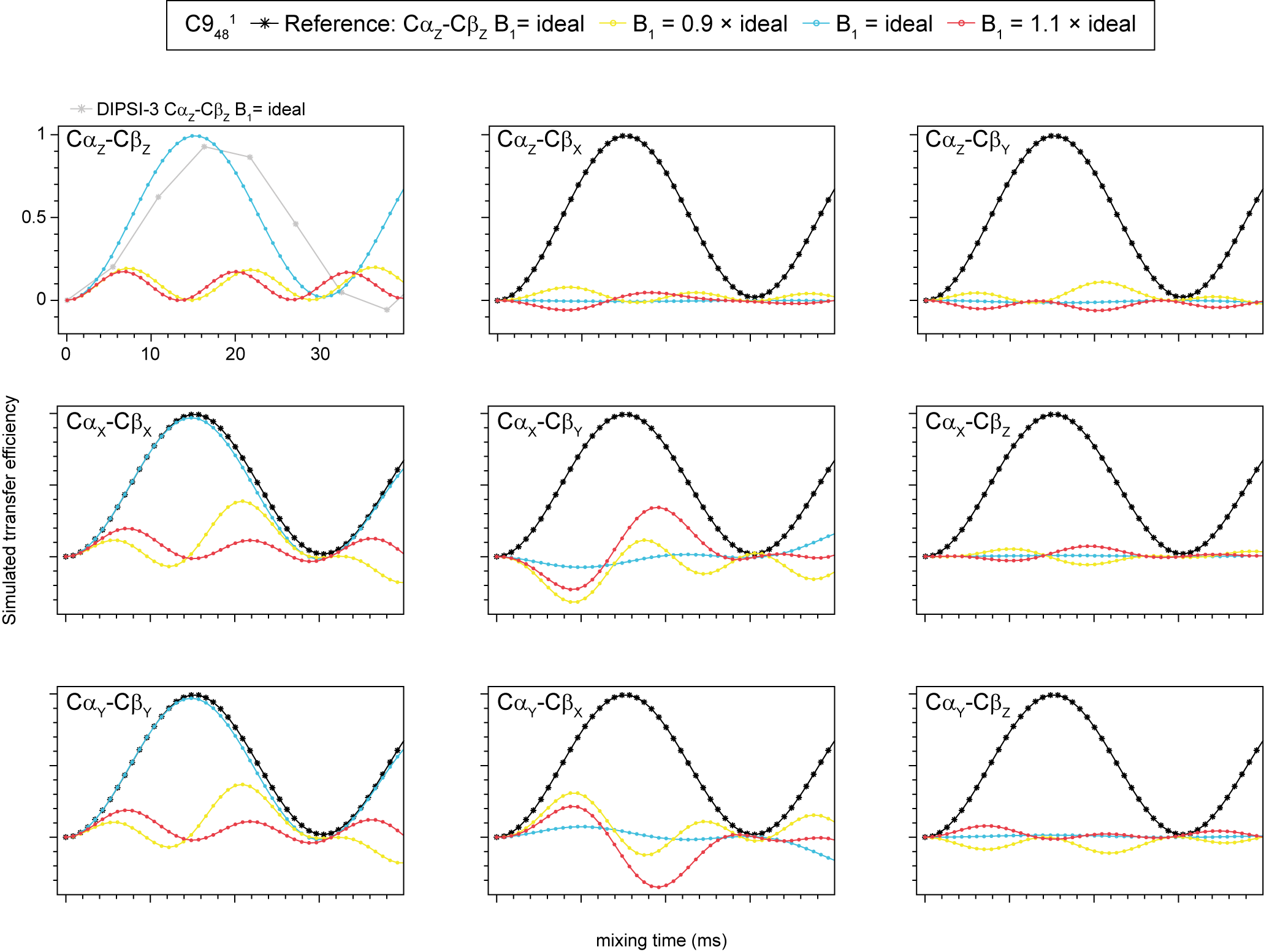


Figure S 5. Simulations of polarization transfer dynamics between different components of two ^13^C spins representing Cα and Cβ nuclei for C9_48_^1^ mixing^8^. The nutation frequency was set to 20.625 kHz (blue lines), corresponding to 0.375 times the magic-angle spinning rate. The effect of radio-frequency inhomogeneity was simulated by using non-ideal conditions (yellow and red lines).


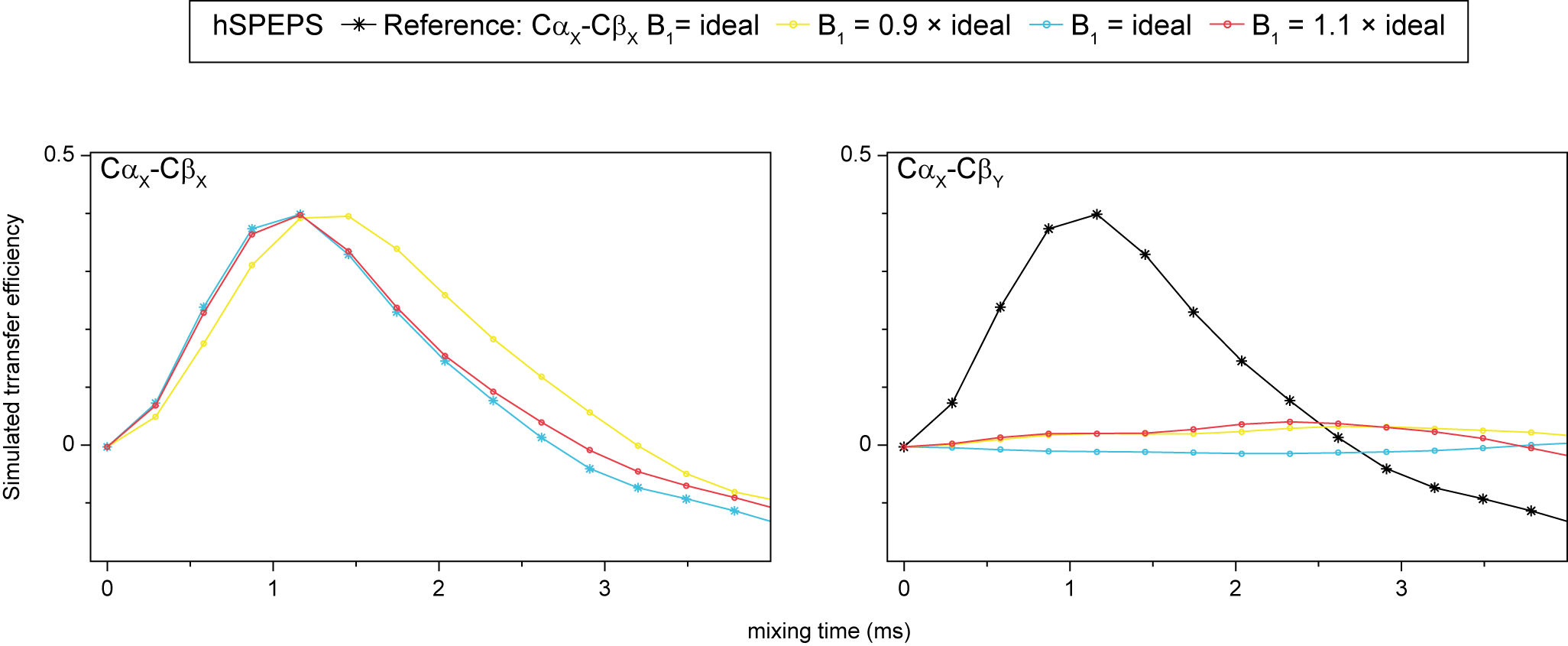


Figure S 6. Simulations of polarization transfer dynamics between different components of two ^13^C spins representing Cα and Cβ nuclei for hSPEPS mixing^7^. The average nutation frequency was set to 27.5 kHz (blue lines), corresponding to 0.5 times the magic-angle spinning rate. The nutation frequency was switched between 1.25 and 0.25 times the magic angle spinning rate, following the published description of the mixing sequence^7^. The effect of radio-frequency inhomogeneity was simulated by using non-ideal conditions (yellow and red lines).


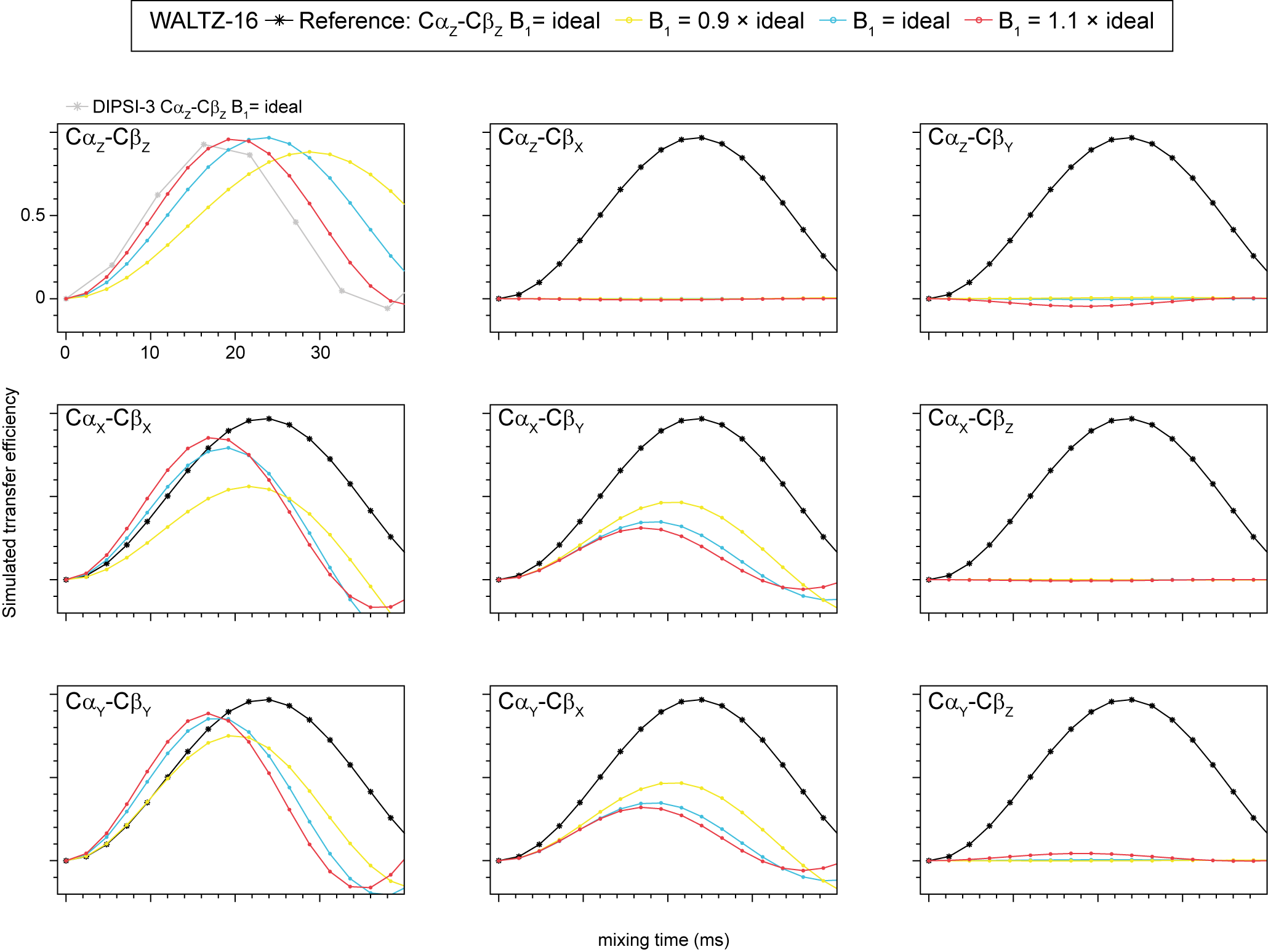


Figure S 7. Simulations of polarization transfer dynamics between different components of two ^13^C spins representing Cα and Cβ nuclei for WALTZ-16 mixing. The nutation frequency was set to 10 kHz (blue lines). The effect of radio-frequency inhomogeneity was simulated by using non-ideal conditions (yellow and red lines).


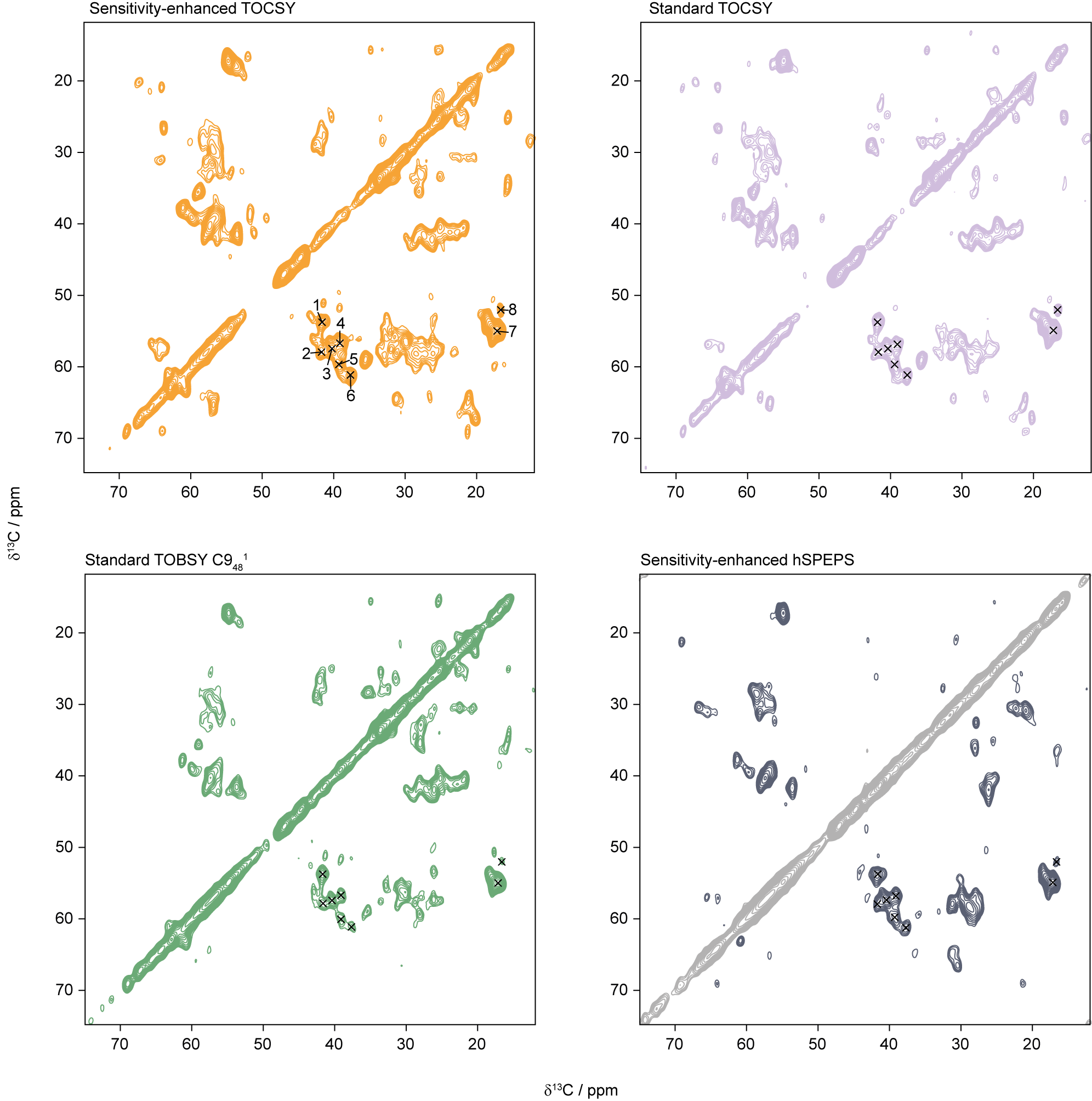


Figure S 8. ^13^C detected 2D hCC spectra of GlpG recorded using different ^13^C-^13^C mixing sequences. These are the full versions of the spectra for which extracted regions are shown in figure 2 in the main article. Each spectrum was recorded using 400 scans and 80 points in the indirect dimension, corresponding to an indirect acquisition time of 4.2 ms. The total experimental time for each spectrum was 10 hours.


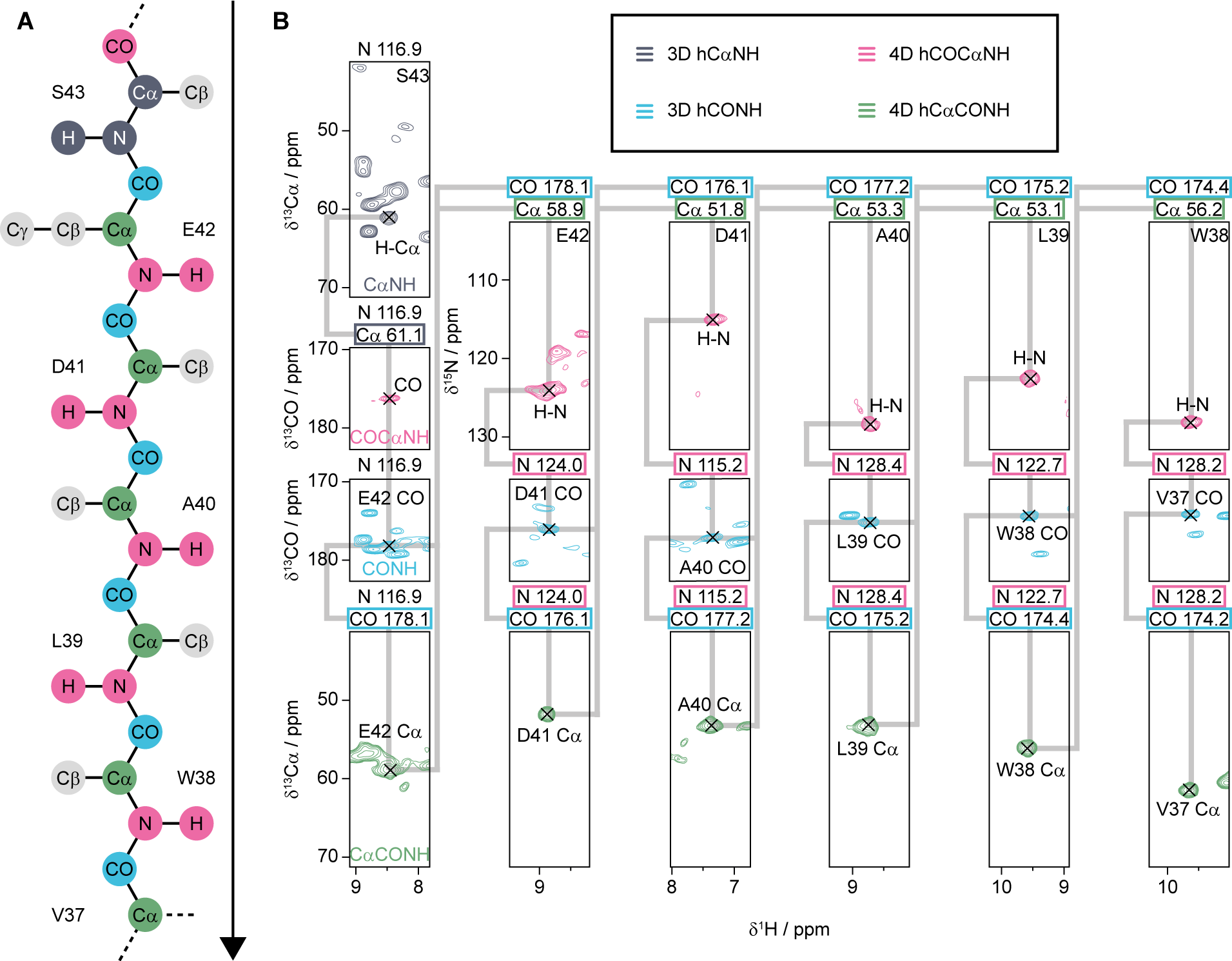


Figure S 9. Example of backbone assignments based on sensitivity-enhanced 3D (hCANH (dark grey), hCONH (blue)) and 4D (hCACONH (green), hCOCANH (pink)) spectra. (A) Scheme of the backbone walk for residues V37 to S43 with each atom coloured according to which spectrum it is identified in. (B) Strip plots showing the backbone walk starting from the Cα-N-H peak of residue S43.


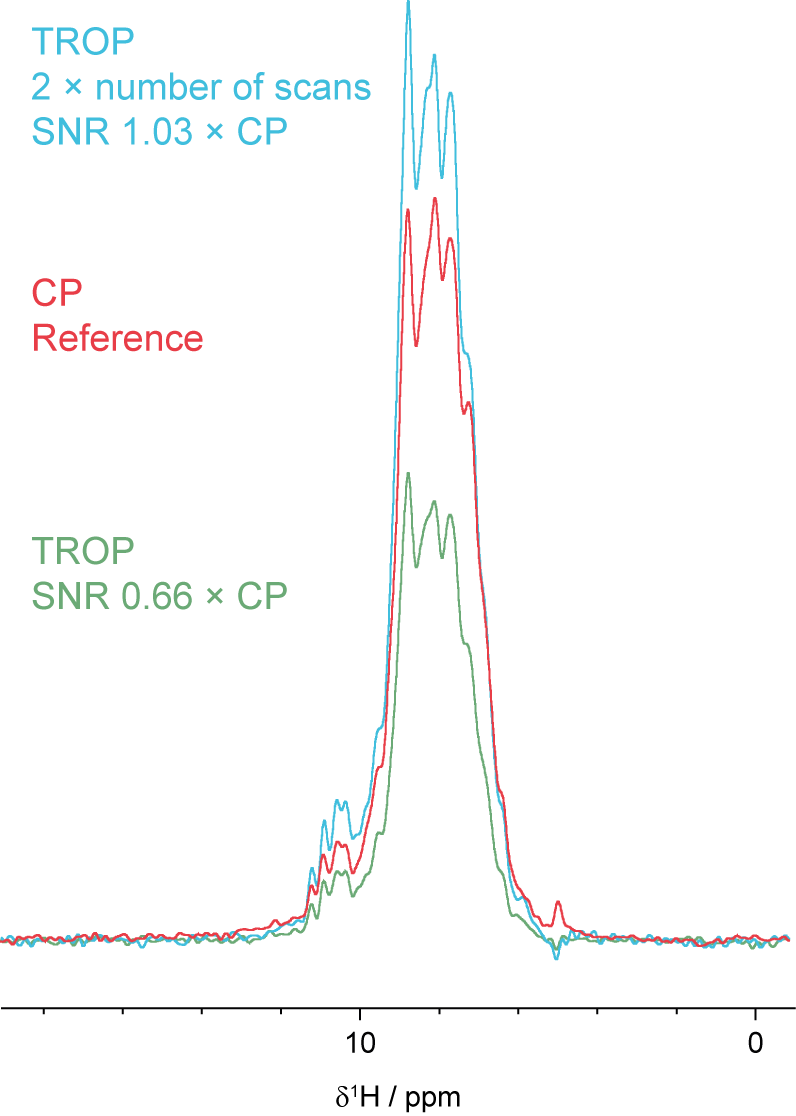


Figure S 10. Comparison of ^1^H detected hnH 1D spectra using CP or TROP for the ^15^N-^1^H magnetization transfer step. CP was used for the ^1^H-^15^N magnetization transfer step in both approaches. The red and green spectra were recorded with 64 scans and the blue spectrum with 128 scans. All spectra were processed using an exponential window function with a line broadening of 20 Hz. The signal-to-noise ratios were extracted from the spectra in TopSpin 4.


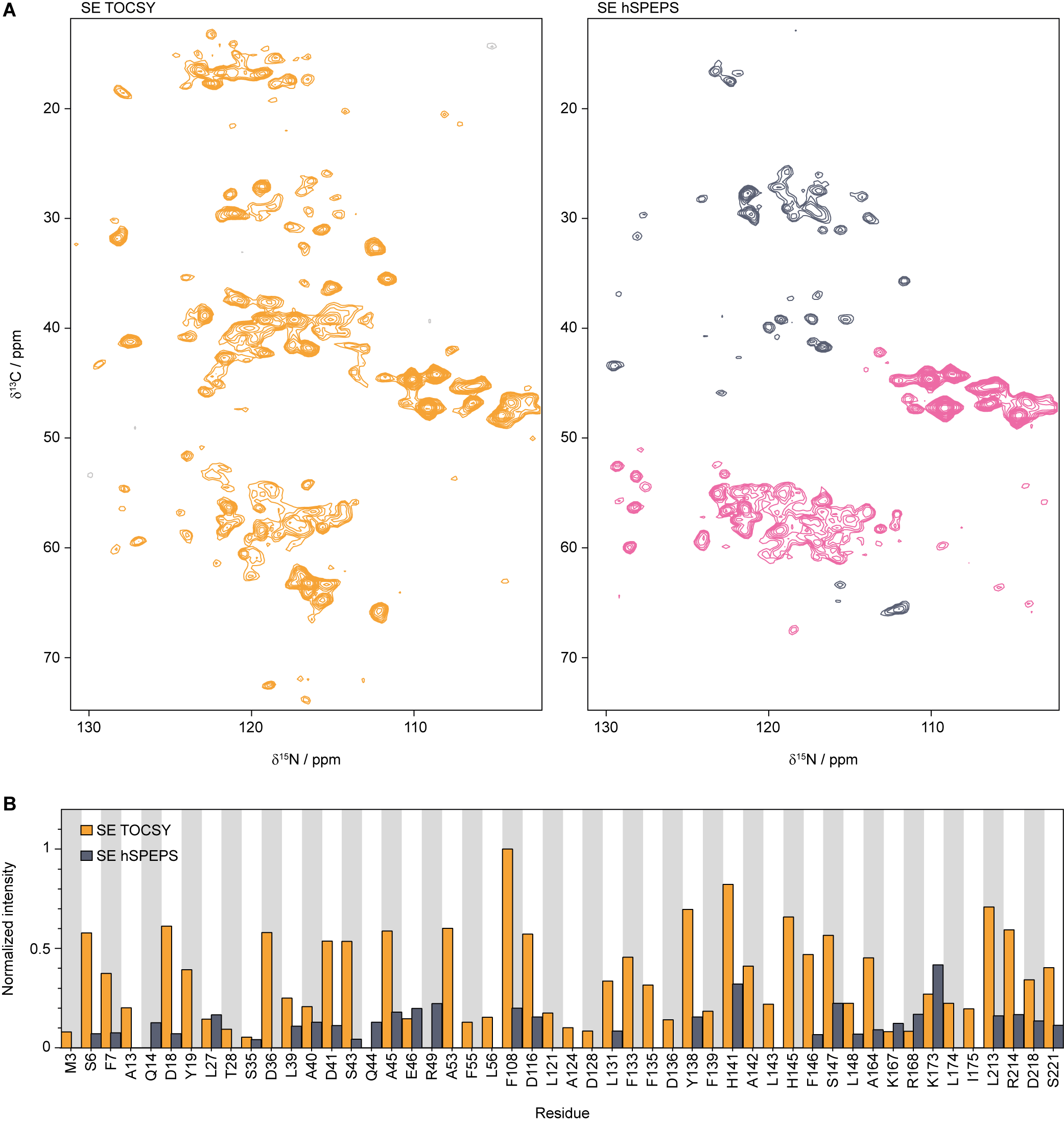


Figure S 11. Comparison of ^1^H detected double sensitivity-enhanced 3D hCXcaNH spectra using zTOCSY (orange) and hSPEPS (dark gray/pink) for ^13^C-^13^C transfers. (A) 2D NCX projections (left zTOCSY, right hSPEPS). (B) Bar plot showing the peak intensities for all isolated Cβ-N-H peaks in the 3D spectra. Orange bars represent peaks in the zTOCSY spectrum and dark gray bars represent peaks in the hSPEPS spectrum. The peak intensities are normalized to the most intense peak (F108 Cβ-N-H in the zTOCSY spectrum). The spectra were recorded at 55 kHz MAS on a 600 MHz spectrometer. Each 3D was recorded in ca 34 hours with 25 % NUS and reconstructed and processed using nmrPipe.

**References**

1. Bak, M., Rasmussen, J. T. & Nielsen, N. C. SIMPSON: A General Simulation Program for Solid-State NMR Spectroscopy. *J. Magn. Reson.* **147**, 296–330 (2000).

2. Delaglio, F., Grzesiek, S., Vuister, G., Zhu, G., Pfeifer, J. & Bax, A. NMRPipe: A multidimensional spectral processing system based on UNIX pipes. *J. Biomol. NMR* **6**, (1995).

3. Blahut, J., Brandl, M. J., Pradhan, T., Reif, B. & Tošner, Z. Sensitivity-Enhanced Multidimensional Solid-State NMR Spectroscopy by Optimal-Control-Based Transverse Mixing Sequences. *J. Am. Chem. Soc.* **144**, 17336–17340 (2022).

4. Zhou, Z., Kümmerle, R., Qiu, X., Redwine, D., Cong, R., Taha, A., Baugh, D. & Winniford, B. A new decoupling method for accurate quantification of polyethylene copolymer composition and triad sequence distribution with 13C NMR. *J. Magn. Reson.* **187**, 225–233 (2007).

5. Rienstra, C. M., Zhou, D. H. & Rienstra, C. M. High-performance solvent suppression for proton detected solid-state NMR. *J. Magn. Reson.* **192**, 167–72 (2008).

6. Blahut, J., Brandl, M. J., Sarkar, R., Reif, B. & Tošner, Z. Optimal control derived sensitivity-enhanced CA-CO mixing sequences for MAS solid-state NMR – Applications in sequential protein backbone assignments. *J. Magn. Reson. Open* **16**–**17**, 100122 (2023).

7. Nimerovsky, E., Kosteletos, S., Lange, S., Becker, S., Lange, A. & Andreas, L. B. Homonuclear Simplified Preservation of Equivalent Pathways Spectroscopy. *J. Phys. Chem. Lett.* **15**, 6272–6278 (2024).

8. Tan, K. O., Agarwal, V., Lakomek, N.-A., Penzel, S., Meier, B. H. & Ernst, M. Efficient low-power TOBSY sequences for fast MAS. *Solid State Nucl. Magn. Reson.* **89**, 27–34 (2018).
